# Mesenchymal stromal/stem cell-derived extracellular vesicles in tissue repair: challenges and opportunities

**DOI:** 10.7150/thno.40122

**Published:** 2020-05-01

**Authors:** Suzy Varderidou-Minasian, Magdalena J. Lorenowicz

**Affiliations:** 1 Center for Molecular Medicine, University Medical Center Utrecht, Universiteitsweg 100, 3584 CG, Utrecht, The Netherlands; 2 Regenerative Medicine Center, Uppsalalaan 8, 3584 CT, Utrecht, The Netherlands

**Keywords:** mesenchymal stromal/stem cells, extracellular vesicles, isolation, *in vivo*, tissue repair

## Abstract

Mesenchymal stem/stromal cells (MSCs) are important players in tissue homeostasis and regeneration owing to their immunomodulatory potential and release of trophic factors that promote healing. They have been increasingly used in clinical trials to treat multiple conditions associated with inflammation and tissue damage such as graft versus host disease, orthopedic injuries and cardiac and liver diseases. Recent evidence demonstrates that their beneficial effects are derived, at least in part, from their secretome. In particular, data from animal models and first-in-man studies indicate that MSC-derived extracellular vesicles (MSC-EVs) can exert similar therapeutic potential as their cells of origin. MSC-EVs are membranous structures loaded with proteins, lipids, carbohydrates and nucleic acids, which play an important role in cell-cell communication and may represent an attractive alternative for cell-based therapy. In this article we summarize recent advances in the use of MSC-EVs for tissue repair. We highlight several isolation and characterization approaches used to enrich MSC-derived EVs. We discuss our current understanding of the relative contribution of the MSC-EVs to the immunomodulatory and regenerative effects mediated by MSCs and MSC secretome. Finally we highlight the challenges and opportunities, which come with the potential use of MSC-EVs as cell free therapy for conditions that require tissue repair.

## Introduction

Mesenchymal stem/stromal cells (MSCs) are multipotent cells capable of differentiating into multiple lineages of the mesenchyme. They can be isolated from a variety of tissues including the bone marrow, adipose tissue, the placenta and cord blood 1. MSCs express markers such as CD73, CD90 and CD105 and are negative for haematopoietic and endothelial markers (CD14, CD11b, CD19, CD79α, CD34, CD45, HLA-DR) 2. The discovery that MSCs are immunoregulatory and have regenerative properties has attracted significant clinical interest with MSCs being used for cell therapy since the early 2000s. Although there are currently over 600 ongoing registered clinical trials using MSCs (www.clinicaltrials.gov), the molecular mechanism underlying the beneficial effects of MSC in tissue injury and inflammation remains poorly understood. A growing body of evidence suggests that therapeutic efficacy of MSC therapy is not dependent on the engraftment of MSCs at the site of injury or the differentiation capability of the transplanted MSCs 3-8, but relies on their paracrine signaling. Recent studies have demonstrated that MSC-derived extracellular vesicles (MSC-EVs) exert beneficial effects in different disease models including myocardial ischemia/reperfusion injury, skin wound healing, kidney injury, graft versus host disease, stroke and sepsis 9-12.

**Figure 1:**
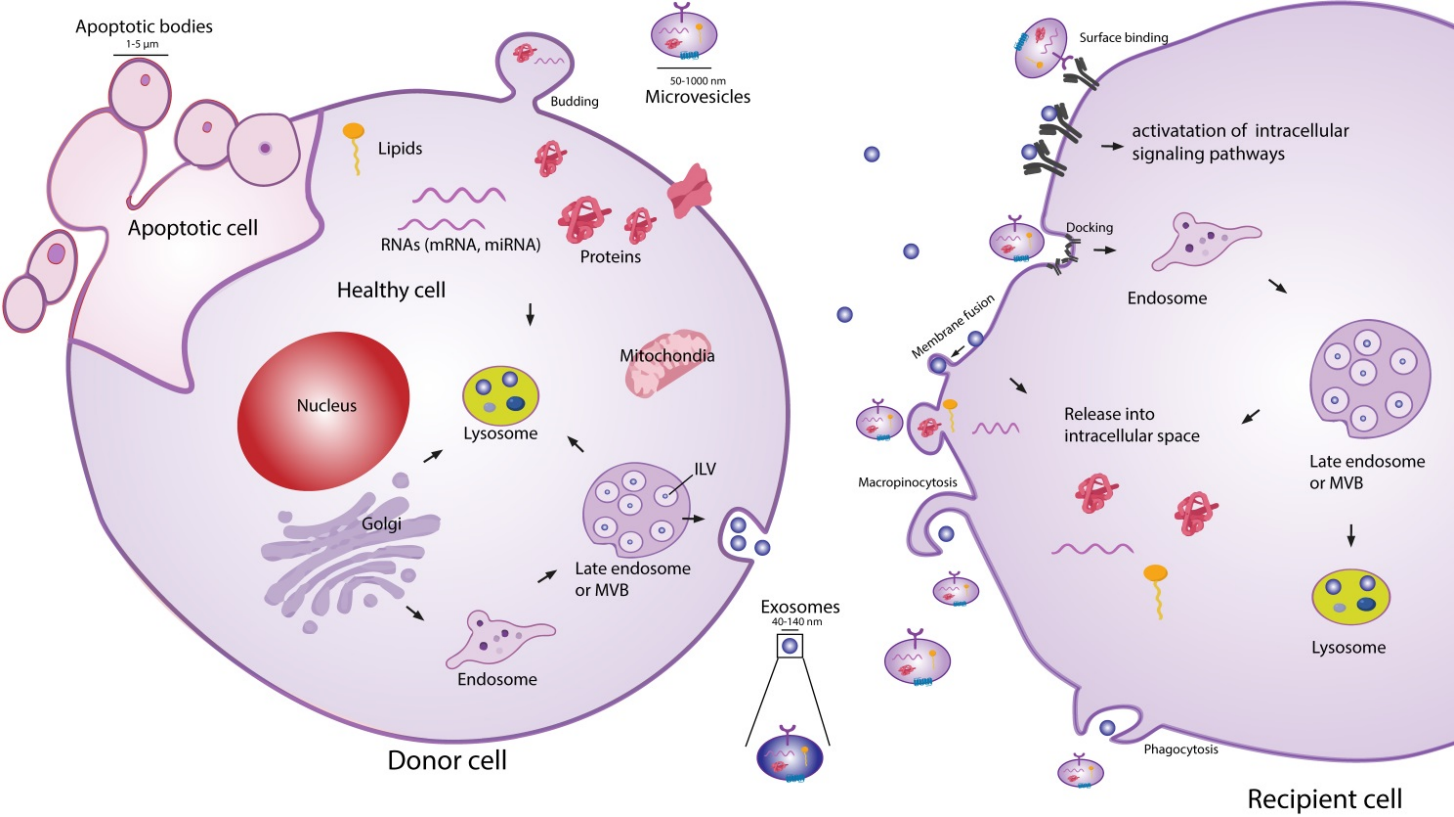
** Schematic representation of EV biogenesis, secretion and uptake.** Exosomes (40-140 nm) are intraluminal vesicles (ILV) formed by the inward budding of endosomal membrane during maturation of multivesicular body (MVB), which are secreted upon fusion of the MVBs, with the plasma membrane. Microvesicles (50-1000 nm) comprise large and heterogeneous group of vesicles with different membranes depending on their origin and morphology. Apoptotic bodies are shedding vesicles derived from apoptotic cells. After the release into the extracellular space, EVs can bind to the cell surface receptors and can initiate intracellular signaling pathways. EVs can also be internalized through processes such as macropinocytosis, phogocytosis or can fuse with the plasma membrane and release their content in the intracellular space. The cargo consisting of proteins, RNA's and lipids are released in the intracellular space or taken up by the ensosomal system of the recipient cell.

Extracellular vesicles (EVs) are secreted membranous structures, covering various subtypes such as exosomes, microvesicles or apoptotic bodies (**Figure 1**). Exosomes are the smallest secreted vesicles (40-140nm). They are formed by the invagination of the membrane of the multivesicular bodies (MVBs) from the cellular endo-lysosomal system^13^-^15^. Upon fusion between MVBs and the plasma membrane, exosomes are released into the extracellular environment. Another subpopulation of EVs are microvesicles (MVs; 50-1000 nm), which bud directly off the plasma membrane 13,15,16. When cells are undergoing apoptosis, they release larger MVs in the form of apoptotic bodies (1-5 µm). EVs are very heterogeneous in size and content, and due to a lack of reliable tools and specific markers to distinguish EV subtypes, good classification of exosomes and MVs is an ongoing challenge 17,18. Similarly to EVs from other cell types, MSC-EVs can be the best characterized according to guidelines of the Minimal Information for Studies of EVs (MISEV 2018 17,19).

EVs exert many of their functions acting as an intercellular shuttle, transporting cargo such as protein, RNA, lipids and carbohydrates between cells. The specific cargo composition of EVs is largely defined by the tissue/cell type they originate from 20,21. The reports on beneficial effects of MSC-EVs in inflammation and tissue repair have triggered a significant interest into the application of MSC-EVs as a cell-free therapy. MSC-EVs-based therapy has several advantages over cellular therapies. EVs as a therapeutic option should not be as susceptible as MSCs to undesirable changes resulting from injection into the inflammatory environment of injured tissue. Injection of EVs also carries a lower safety risk, as they cannot self- replicate. In contrast to cells, EVs can also be relatively easily and safely genetically manipulated to carry desired therapeutic cargo. Their manufacture and storage is less demanding and likewise less costly than current cellular therapies. Finally, due to their small size compared to MSC, the delivery of EVs by intravenous (IV) injection presents lower risk of vascular obstructions. However, there are still many important questions that remain to be answered, before MSC-EVs can become a fully realized cell free therapy. Here we highlight only few of them:

- What is a relative contribution of MSC-EVs to the therapeutic effect of MSCs?
- How efficient are MSC-EVs when compare to MSCs?
- Is it necessary for MSC-EVs to be targeted to the injured tissues?
- Which MSC-EVs populations are most therapeutically potent?
- What is a molecular mechanism underlying the therapeutic effect of MSC-EVs?

The purpose of this review is to summarize the current state of art of MSC-EVs characterization and therapeutic use and to give an overview of existing evidence, which could help to answer the highlighted questions.

## Isolation, characterization and quantification of MSC-EVs

**Figure 2:**
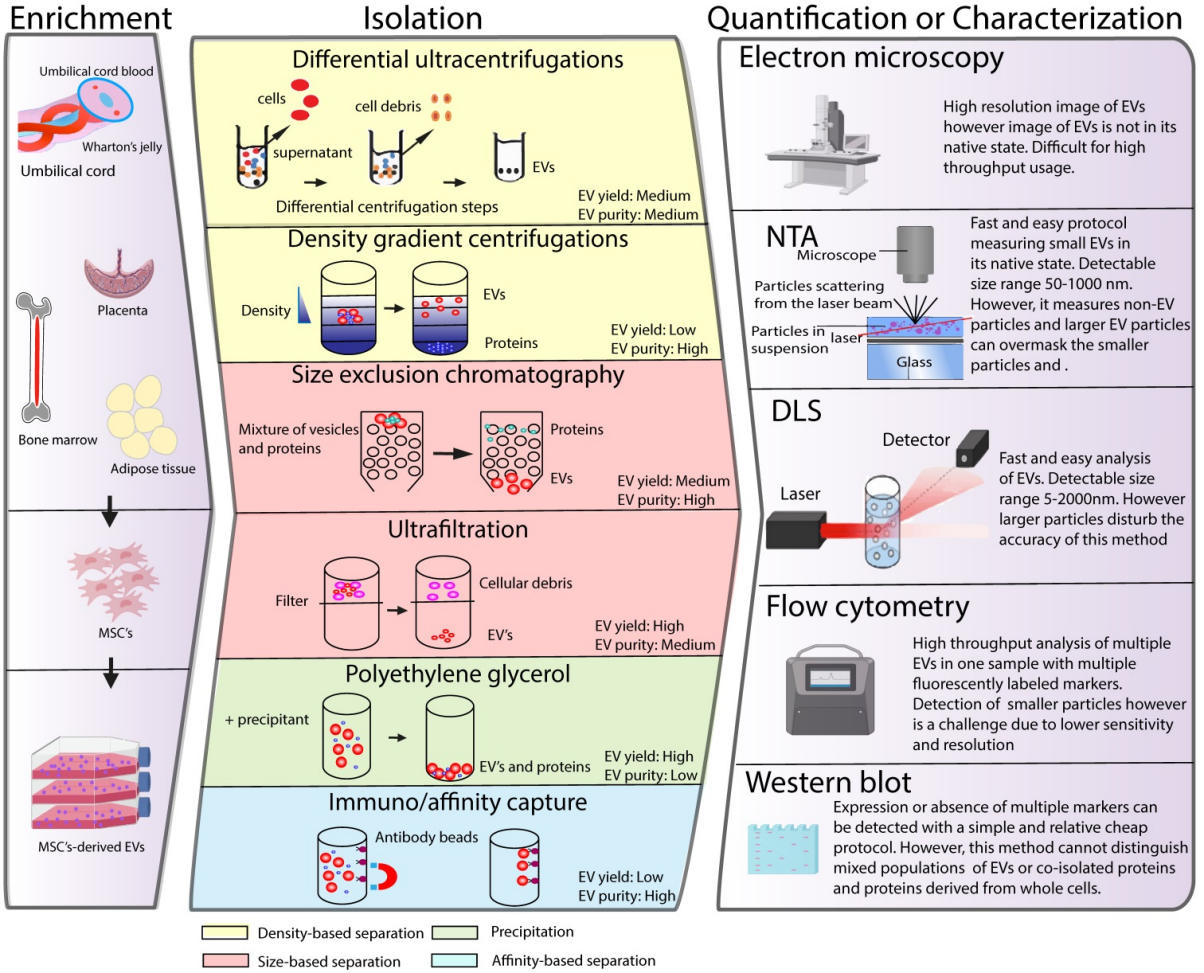
** Schematic classification of common methods of EVs isolation, characterization and quantification.** (Left panel) MSCs can be isolated from various tissues such as umbilical cord, bone marrow, placenta or adipose tissue. MSCs are cultured *in vitro* and the conditioned medium is collected to enrich for EV. Middle panel depicts different strategies for EV isolation and different EV properties used as a base for the isolation protocols are indicated in colours. Right panel illustrates strategies such as electron microspopy, nanoparticle tracking (NTA), dynamic light scattering (DLS), flow cytometry or western blot, which are typically used for EV quantification or characterization.

The application of MSC-EVs as a therapy has generated demand for EV isolation and quantification procedures suitable for a clinical setting. In recent years, a plethora of different techniques have been developed to isolate, characterize and quantify EVs. However, effective isolation, characterization and quantification of these membrane structures remains a challenging task due to their small size and physiochemical heterogeneity. In the following section we summarize different EV isolation and quantification techniques currently used in the field and discuss their suitability for future clinical use **(Figure 2)**. For more detailed information about each of the techniques, we refer the reader to recent reviews on this topic 22-27.

### Isolation of EVs

Typical isolation methods to separate EVs from the rest of the cellular compartment are based on EV properties such as density, size and surface components. Isolation protocols with less steps result in higher EV yield compared to more labor-intense ones, however they deliver EVs of lower purity. 28. The International Society for Extracellular Vesicles recommends combining different isolation approaches to ensure the highest EV yield and purity.

#### Differential ultrahigh-speed centrifugation

Differential ultracentrifugation is the most common method utilized for smaller EV isolation. This was also the most generally used method in the pre-clinical studies testing the therapeutic potential of MSC-EVs in tissue repair *in vivo* (see **Table 1**). This technique uses series of differential centrifugation steps to remove cells and large cellular debris and precipitates EVs at high speed. Larger particles remain in the supernatant whereas smaller EVs are pelleted 29. The isolation of EVs with this method results in medium yield and purity of EVs. The primary disadvantages of this method are that it is a time-consuming process that requires the use of expensive equipment, currently making it unsuitable for the clinical setting. Furthermore, the isolated EV population can be contaminated with proteins, and the integrity of the EVs may be compromised due to the high centrifugation speed.

#### Density gradient centrifugation

An alternative method to separate the smaller EVs is the density gradient centrifugation, which is based on different floating densities. Sucrose or iodixanol solutions with different densities are preloaded into a centrifuge tube with the sample, which is followed by ultracentrifugation. The EVs float based on their different flotation densities allowing a better separation of EVs from impurities 30-32. As a result, this method delivers EVs with relatively high purity. Similar to differential ultrahigh-speed centrifugation, this method requires expensive equipment and is time consuming. Due to the different centrifugation steps there is high risk of vesicle loss and damage. In addition, sucrose and iodixanol solutions may negatively influence the functionality of isolated EVs, which in case of MSC-EVs can reduce their therapeutic activity.

#### Size exclusion chromatography

Size-exclusion liquid chromatography (SEC) can separate EVs from proteins based on their size. SEC uses a porous matrix packed into a column that allows the sample to pass through a porous stationary phase of polymers. Smaller sized particles such as proteins will elute later because they are slowed down by entering the pores of the polymer. EVs, which are larger in size than proteins, will elute earlier because they travel more quickly through the column 33. This method was shown to isolate EVs with minimal damage and to preserve their biophysical and bioactive properties 34. Isolation of MSC-EVs by SEC, for example, preserved their inhibitory function on T cell proliferation, which was not the case for MSC-EVs isolated using differential ultracentrifugation steps. This shows that SEC is suitable for efficient separation of relatively pure and functional populations of EVs 35. Additionally, a big advantage of this method is that it is relatively easy to scale up, which is especially important for the future clinical application of MSC-EVs. Drawbacks of this isolation procedure include labor intensity and a high chance of sample contamination with protein aggregates and lipoproteins 36.

#### Ultrafiltration

Ultrafiltration is a method used to isolate EVs based on their size. It employs membrane filters with different pore sizes allowing smaller particles to penetrate and pass through the membrane while larger particles are excluded. Different membranes are sequentially used to first remove cells and debris. Several studies have demonstrated therapeutic activity of MSC-EVs isolated using ultrafiltration 37,38. A recent report, which compared the use of ultrafiltration with ultracentrifugation to purify MSC-EVs, demonstrated that the ultrafiltration procedure enriches for larger EVs compared to using ultracentrifugation 39. In addition, ultrafiltration was more efficient in removing the smaller sized proteins from the EV suspension, while with ultracentrifugation; these proteins were pelleted with the EVs. Another report compared ultrafiltration, ultracentrifugation, and precipitation methods, and evaluated the purity and yield of isolated EVs using NTA 40. Isolation of EVs by ultrafiltration resulted in a 50-fold increase in yield compared to ultracentrifugation and in a 20-fold increase compared to precipitation methods. Therefore, especially for future therapeutic use of MSC-EVs, ultrafiltration is a time and cost-effective alternative to the gold-standard ultracentrifugation method. One of the disadvantages of ultrafiltration is that the membrane pores can be easily blocked leading to low EV yield. Furthermore, a force is applied to pass the sample through the membrane, which might lead to vesicle damage. More recently, tangential flow filtration (TFF) was developed, which is a form of ultrafiltration applied in a cross-flow (tangential) mode, to separate EVs from proteins 41. It allows the fluid to flow tangentially across the membrane surface, instead of vertically in the conventional dead-end filtration, therefore avoiding clogging the membrane pores. Thus, TFF might serve as a better alternative to the conventional ultrafiltration in the clinical use of MSC-EVs.

Ultrafiltration is often combined with SEC (referred as UF-LC) to further improve EV separation. The limited volume (from 0.5 µl up to 2 ml) for SEC that can be loaded on the column, can be resolved by using ultrafiltration prior to SEC. The UF-LC allows isolation of EVs with high purity and was shown to be relatively simple and can be automated. Compared to UC isolation method, the UF-LC was shown to deliver higher EV yields as evaluated by NTA and more intact and pure vesicles 42.

#### Polyethylene glycol

Polyethylene glycol (PEG) is a method to isolate EVs by precipitation. PEG, which is water-excluding precipitant, is added to the sample followed by an incubation step and centrifugation to concentrate the particles. There are commercially available kits using PEG to isolate EVs such as ExoQuick or Total Exosome Isolation Kit. PEG-based approaches allow the isolation of EVs from cell culture conditioned medium with high EV yield and recovery 43. A recent study demonstrated that PEG -based EV purification may also better preserve the association of proteins bound to the EV surface when compared to ultracentrifugation method, which is important for downstream analysis of EV functionality. MSC-EVs carrying Wnt3a protein on their surface isolated using PEG-based approach stimulated dermal fibroblasts migration and proliferation and endothelial angiogenesis in a Wnt3a-dependent manner more efficiently then MSC-EVs isolated by differential centrifugation 44. Overall PEG-based methods are user friendly and cheap, however, it is important to consider that the precipitated sample is low in purity because of contaminants, which co-precipitate with EVs such as protein aggregates. This might have an impact on the EV functionality and therapeutic efficacy.

#### Immuno/affinity capture

Immuno/affinity capture-based methods separate EVs based on their surface protein expression. A priori knowledge of markers expressed on EVs is therefore needed for their isolation. Members of the tetraspanin family (CD9, CD63 and CD81), which are expressed on the membranes of EVs are used for the enrichment. This is often followed by the use of antibody-coated magnetic beads to isolate the EVs with high purity 45. Although this method was shown to be suitable to specifically capture EVs with great recovery, the binding is not easily reversible making it challenging for *in vivo* studies. Another limitation of this approach is that it cannot be used to isolate EVs from samples with large volume. For this, samples need to be pre-concentrated using ultracentrifugation steps. In addition, with the immuno/affinity capture only a subset of EVs can be isolated, which may only be an advantage for clinical application of EVs, if the subset of EVs with therapeutic properties is well defined. However, the identification of a subset of MSC-EVs with the best therapeutic potential is still an ongoing challenge.

### Characterization and quantification of EVs

Several characterization and quantification methods have been developed to analyze EVs (**Figure 2**), however no single approach allows accurate analysis of EVs. Therefore, multiple techniques are usually utilized to evaluate EV properties. Below we briefly describe these techniques and discuss their suitability in the (pre)clinical setting.

#### Nanoparticle tracking analysis

Nanoparticle tracking analysis (NTA) is a method used to determine the size distribution and concentration of the EVs (particles per mL). This technique is based on a laser light microscopy to measure the light, which is scattered by the individual particles. The motion of the particle relates the rate of Brownian motion to particle size which is tracked by a camera 46. Although NTA is widely used to quantify EVs, this method is very sensitive to any non-EV particle contamination. This might be problematic for quantification of samples of lower purity. In addition, one has to take into account that particles larger than 100 nm tend to be overestimated because these particles can scatter multiple points of light and can therefore be measured as multiple events. These two disadvantages of the method greatly influence the accuracy of EV quantification, which is crucial in future clinical use of EVs. In the studies using EV preparations containing larger sized EVs, the information only on particle concentration with no cell equivalent given is not sufficient and might lead to lack of reproducibility^47^-^50^. The mentioned drawback of NTA might have accounted for the apparent differences in the amount of MSC-EVs administered in the pre-clinical studies described in this review. As shown in **Table 1,** the amount of EVs injected intravenously, in different mouse models of tissue injury, varied from 1.6 x 10^7^ to 2.4 x 10^11^ particles, which is a 15000 fold difference.

Fluorescence NTA is a recent development in NTA, which allows tracking only fluorescently labelled EVs and distinguishing EVs from the rest of the particles/proteins. However, this requires an extra labeling step, which might not be so convenient in the clinical setting. Additionally, labeling restricts the EV detection to specific EV subsets positive for the markers used.

#### Dynamic light scattering

Dynamic light scattering (DLS) is another method to quantify EVs in suspension. Similar to NTA, DLS measures the movement of particles undergoing Brownian motion in suspension. A scattered light from the particles is interfered and the dynamic information is traced and converted using Strokes-Einstein equation 51. With this, the concentration and hydrodynamic diameter can be calculated. DLS can detect small particles (>5 nm) and is best suited for measuring monodisperse particles. Although DLS is simple and fast to use, it detects all scattering particles, and as a consequence, the presence of a few larger particles can mask the smaller ones. Therefore, DLS has limited utility for complex samples including these of lower purity.

#### Electron microscopy

Scanning or transmission electron microscopy, SEM or TEM respectively, are the most commonly used techniques to characterize the microstructure of MSC- EVs. In SEM, the topography of the EV surface is scanned while in TEM, which is more often used, a 2D image of the EV is created with inner structural information. An advantage of SEM and TEM-based imaging, is that it can be combined with immunogold labelling. A specific molecule of interest can be stained using antibody-based approaches, and its localization on EVs can be detected 52. One of the challenges for both techniques however is the low throughput capacity due to the lengthy sample preparation and sample loss 53. Furthermore, the sample processing here results in shrinkage of EV size, resulting in underestimation of EV actual size, as also shown for MSC-EVs 54. The quantification of EVs with this method is possible but is very labor intensive and is, therefore not attractive for use in clinics. Next to SEM and TEM, cryo-electron microscopy (cryo-EM) is also used to analyze the structure and size of EVs. The advantage of cryo-EM is that it does not use staining or chemical fixation procedures and samples are directly applied onto an EM grid, vitrified and visualized. This allows for characterization of EVs near their native state. Another advantage of this method is that it does not require large amount of EVs in the preparation to perform accurate analysis of their size and morphology 55.

#### Protein quantification

A commonly used method to quantify MSC-EVs before use in the *in vivo* studies is determination of the total protein content using biochemical methods such as micro-Bicinchoninic acid or Bradford assay. A drawback here is that protein contaminants co-isolated with EVs can influence the accuracy of the measurement. In the *in vivo* studies testing MSC-EVs in different pre-clinical models, which are evaluated in this review (see **Table 1**), there is substantial variability in the amount of EVs used as measured by the protein content. When considering only the studies in different mouse models of tissue injury, in which EVs were injected IV the amount of applied EVs varies from 250 ng to 200 µg material. This is an 800-fold difference. When the amounts of administered EVs are compared in the models of autoimmune disorders, the difference in the amount of injected EVs is 750-fold. For the liver injury mouse models this difference is 300-fold (IV injections). Since all the studies summarized in this review reveal a positive effect after MSC-EVs treatment, the findings need to be carefully interpreted.

## Relative contribution of MSC-EVs to therapeutic effect of MSCs

The amount of studies demonstrating therapeutic potential of MSC-EVs in different disease models is growing rapidly. However, few test their therapeutic effect in direct comparison to their cellular counterparts, which seems relevant when considering future clinical application of MSC-EVs. The studies that did compare the two in a quantitative manner report different outcomes depending on the disease model in which the MSC-EVs were tested and the type of EVs used. Kim *et al.* demonstrated that EVs were more potent than the MSCs they were isolated from in reducing the inflammation in traumatic brain injury mouse models after single IV injection 56. In contrast, in the collagenase-induced osteoarthritis (OA) model, small EVs (pelleted at 100,000 g by ultracentrifugation) had similar effect as MSCs in protecting mice from osteoarthritic damages, but larger EVs (pelleted at 18,00g) were less potent after single intraarticular administration 57. Also in the acute kidney injury mouse models and in the lung ischemia- reperfusion injury mouse models, small EVs performed equally well as their parental cells in regenerating tissue or preventing tissue damage respectively, after only a single injection 58,59. Ruenn Chai Lai *et al.* further explored the importance of MSC-EVs in tissue repair in myocardial ischemia/reperfusion injury 10. Previously, they showed that the MSC-derived conditioned medium (CM) had cardio protection effect during injury, which was then demonstrated to be mediated by the EVs 60. They concluded that EVs are equally efficient as CM in reducing myocardial ischemia-reperfusion in mice. Thus, the data from the above studies demonstrated that EVs may have major contribution to the paracrine effect of MSCs on tissue regeneration. They also indicate that, at least in the disease models used in these reports, a single injection of EVs has the same therapeutic potential as MSCs themselves. This is promising for the future clinical application of MSC-EVs, as single administration of MSC-EVs limits burden for patients and also lowers the cost of future MSC-EVs based therapy. However, the quantitative comparison of MSC versus MSC-EVs effects presented in the above studies still needs to be taken with caution considering current limitations of the available quantification methods of EVs. Also the amount of EV released by MSCs in these studies was estimated based on the data from two dimensional (*2D*)* in vitro* culture and it is difficult to predict how this compares with amount of EVs that MSCs release *in vivo.*

In recent years several groups have invested in new approaches to culture MSCs in a three-dimensional (3D) manner to better mimic *in vivo* conditions and possibly increase the yield of MSC-EVs produced by these cells, whilst maintaining or even enhancing their therapeutic effect. These 3D systems use a hydrogel containing extracellular matrix (ECM) components. The ECM can also come in a form of a porous scaffold mimicking even better the *in vivo* environment. Qazi *et al*. showed that MSCs grown in 3D produced more cytokines and growth factors then cells propagated in 2D cultures 61). Ni Su *et al*. compared different structural patterns of the ECM (oriented fibers vs not oriented) and showed that in ECM with oriented fibers, MSCs increased the production of anti-inflammatory and pro-angiogenic factors 62. This clearly indicates that culture conditions influence the composition of MSC secretome quantitatively and qualitatively, which should be taken in consideration when choosing the MSC expansion methods for future production of MSC-EVs for clinical use. Recent developments in large-scale MSC expansion for MSC-EVs production include bioreactors such as spinning flask or hollow-fibre 63.

## The mechanism of action behind the therapeutic effect of MSC-EVs

**Figure 3:**
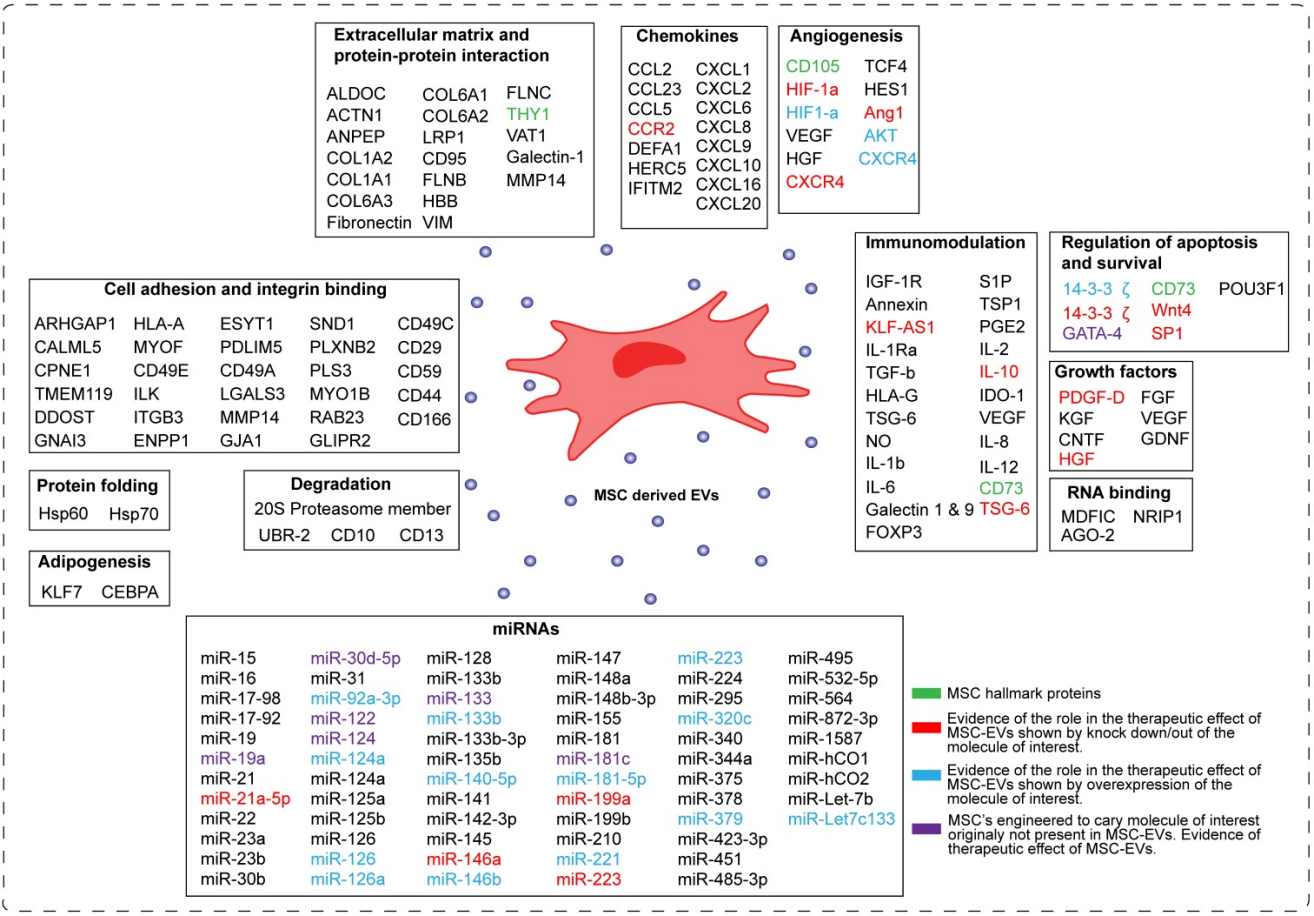
** Schematic representation of the components of MSC-derived EVs.** The molecules present in MSC-EVs can be categorized into sixteen groups based on their molecular and cellular function. These are: -transcription factors, -extracellular matrix proteins, -chemokines, cytokines, -enzymes, -growth factors, RNA binding molecules, -miRNAs, -molecules involved in angionenesis, -cell adhesion, -development, -degradation, -protein folding, -immunomodulation, -regulation of apoptosis and survival, and -adipogenesis. In green are depicted MSC hallmark proteins. In red are depicted molecules, which role in the therapeutic effect of MSC-EVs was proven by knocking them down/out in MSCs. In blue are depicted molecules, which role in the therapeutic effect of MSC-EVs was proven by overexpressing them in MSCs. In violet are depicted molecules, which normally are not present in MSC-EVs, but upon overexpression they induce the therapeutic effect of MSC-EVs.

Despite the increasing interest in the mechanism of action of MSC-EVs, this field is still in its infancy in identifying the molecules responsible for their therapeutic effect. In the Figure 3 we have summarized current understanding of factors involved in the beneficial effects of MSC-EVs. The major processes important for tissue repair and thought to be regulated by MSC-EVs are apoptosis, cell proliferation, angiogenesis and inflammation.

### Bioactive molecules in MSC-EVs identified by omics approaches

Efforts have been made, especially in omics approaches, to identify the relevant bioactive molecules playing a role in the MSC-EVs-mediated tissue repair. A number of RNA-sequencing studies showed that MSC-EVs were selectively enriched for distinct classes of RNAs 64-66. Eirin *et al*. reported that MSC-EVs preferentially express mRNA for transcription factors and genes involved in angiogenesis and adipogenesis 66. In addition, they demonstrated using gene ontology analysis that miRNAs enriched in MSC-EVs such as miR148a, miR532-5p, miR378, and let-7f target transcription factors and genes that participate in several cellular pathways, including angiogenesis, cellular transport, apoptosis, and proteolysis.

A number of groups compared the proteome of MSC-EVs with the proteome of MSCs using mass spectrometry-based proteomic approaches 67-71. Proteins identified in MSC-EVs as well as in their parental MSC, are involved in processes including self-renewal, differentiation and cell proliferation. By comparing the proteome of MSC-EVs and MSCs, unique proteins were identified to be enriched in MSC-EVs 72. These proteins were involved in angiogenesis, apoptosis, inflammation and extracellular matrix remodeling, and several of these factors were reported to be specific for MSC-EVs 73.

It is important to consider that conditions, under which MSCs were cultured such as hypoxia or ischemic and inflammatory conditioning might affect the MSC-EVs content and properties 74-76. In addition, EVs derived from different sources of MSCs display different transcriptome and proteome profiles 77. This should be taken into scrutiny when designing the strategy for MSC-EVs production for their future therapeutic use.

### MSC-EVs and immunomodulation

MSC-EVs are immunologically active and contain molecules that can modulate the immune cells. In Figure 3 we depict potential effectors identified within MSC-EVs that show immune-modulatory properties. Among others, MSC-EVs contain chemokines and inflammatory cytokines that can modulate both innate (natural killer cells, dendritic cells and macrophages) and adaptive immune cells (B and T cells) 11,78.

A number of research groups used overexpression and knock down experiments to identify the bioactive immunomodulatory molecules responsible for therapeutic effect of MSC-EVs 12,79,88-97,80,98-105,81-87. For example, Eirin *et al*. showed that MSC-EVs containing IL-10 improved renal structure and function, decreased renal inflammation and increased the number of reparative macrophages in renal artery stenosis (RAS) in pigs 79. These effects were abolished when EVs from MSCs, where IL-10 was knocked down were used for treatment of RAS. Furthermore, MSC-EVs expressing TSG-6 protein, an immunomodulatory molecule induced in pathological conditions in response to increased inflammation, were able to decrease lung inflammation and cell death in bronchopulmonary dysplasia mice. This therapeutic effect was abrogated when MSC-EVs lacking TSG-6 expression were used 107. Another study showed that MSC-EVs containing C-C motif chemokine receptor-2 (CCR2) were able to inhibit the activation of macrophages and monocytes and protect against renal/ischemia injury in mouse 108. Several reports have also demonstrated the immunomodulatory role of specific miRNAs highly expressed in MSC-EVs. For example, MSC-EVs carrying miRNAs such as miR-21-5p, miR-142-3p, miR-223-3p and miR-126-3p regulated dendritic cell maturation and promoted their anti-inflammatory potential 109. Furthermore, MSC-EVs containing miR-223-3p were able to reduce pro-inflammatory cytokine production in macrophages, systemic inflammatory response, cardiac dysfunction and increase survival in polymicrobial sepsis murine model 110. MSC-EVs from miR-223-3p knockout mice failed to show these beneficial therapeutic effects. On the other hand, MSC-EVs overexpressing miR-223 were shown to protect from liver injury in autoimmune hepatitis models and downregulate many inflammatory genes and cytokines 100. MSC-EVs carrying miRNAs such as miR-21a-5p, miR-146a, miR-199a, and miR-223 regulated several inflammatory genes (IL-6, NLRP3) and induced macrophage polarization towards an anti-inflammatory M2 phenotype. Silencing expression of these miRNAs in MSC-EVs resulted in the reduction of their therapeutic effect 111.

In summary, the important component of the mechanism used by MSC-EVs to promote tissue repair is the regulation of the immune system.

### MSC-EVs in regulation of angiogenesis, cell proliferation and survival

MSC-EVs contain growth factors (GDNF, VEGF, FGF etc) and angiogenic factors (HGF, Ang1, HES1, S1P etc), which are known to promote tissue repair and regeneration 112,113. Shuling Hu *et al*. showed that MSC-EVs containing angiopoietin-1 (Ang1), an angiogenesis regulator, restore protein permeability across injured human lung microvascular endothelial cells, while MSC-EVs lacking Ang1 eliminated the therapeutic effect 81. Kai Kang *et al*. demonstrated that EVs derived from MSCs overexpressing CXCR4 restore cardiac function by increasing angiogenesis, reducing infarct size and improving cardiac remodeling 87. This effect was mediated by upregulation of IGF-1α and pAkt levels and downregulation of caspase 3 levels. In addition, they showed that these effects were abolished by CXCR4 knockdown. Overexpression of certain miRNAs in MSCs can also contribute to enhanced therapeutic effects (Figure 3). For example, EVs derived from MSCs overexpressing miR-21 had protective effects in spinal cord injury by targeting several genes involved in the inhibition of cell apoptosis 88. On the other hand, EVs derived from MSCs overexpressing miR-140-5p were able to promote proliferation and migration of articular chondrocytes and prevented OA development in rat model by enhancing SOX9 expression and extracellular matrix (ECM) generation 92.

### Engineered MSC-EVs

MSC-EVs can also be engineered to carry a desired molecule with a therapeutic potential and facilitate the delivery of such a factor to an injured tissue. Several studies have shown therapeutic effects of such engineered MSC-EVs (Figure 3, factors indicated in violet) 89,92,95-97,102,114. For example MSC-EVs engineered to overexpress GATA4, a factor important for regulation of angiogenesis and cell survival, promoted cardiomyocytes survival, reduced their apoptosis and restore cardiac contractile functions in neonatal rat hypoxia model 89. Another example is a study by Jiang et al, which demonstrated that MSC-EVs engineered to overexpress miR-30d-5p, known to regulate autophagy and apoptosis in brain development, prevent brain injury 114. This was mediated by inhibiting autophagy-mediated inflammatory response and promoting microglial polarization.

Taken together, significant effort still needs to be invested in better understanding of the molecular mechanism underlying the therapeutic effects of MSC-EVs. The knowledge gained from these studies will be crucial in designing the most effective MSC-EV-based therapies.

## Localization of injected MSC-EVs in tissues

To understand the mechanism of action behind the therapeutic effect of MSC- EVs, it is important to follow their fate after *in vivo* administration. EVs can be tracked *in vivo* by labeling them directly after isolation or indirectly by transfecting the EVs-secreting cells with vectors containing imaging reporter genes (**Table 2**). In direct labeling, lipophilic tracer dyes (DiR, DiD, PKH), nanoparticles (radioisotopes such as ^99m^Tc- HMPAO, ^111^In-oxine) or membrane permeable chemical compounds (carboxyfluorescein diacetate succinimidyl ester (CFDA-SE), calcein acetomethoxyester (calcein-AM)) have been used to label the EVs after isolation. The direct labeling protocol is relatively simple and inexpensive. Labeling methods using nuclear imaging and magnetic resonance (^111^ In-oxine, ^99mTc^-HMPAO, USPIO) are stable and highly quantitative but require large amount of EVs. In the methods using CFDA-SE and calcein-AM the non-fluorescent dyes enter EVs and once esterase enzymes cleave the acetate group of the dyes, a fluorescent membrane impermeable ester, carboxyfluorescein succinimidyl ester (CFSE) or calcein are produced respectively 115-117. The advantage of this labeling approach over the lipophilic dyes is, that it can be used to discriminate between intact EVs and cell debris.

The indirect labeling strategy uses genetically modified cells, which secrete EVs containing a reporter protein. This reporter protein is inherited upon cell division and can be used for long term isolation of EVs. Fluorescent proteins (GFP, RFP or dTomato) or luciferase enzyme-substrates (Gluc, GlucB or Rluc) generate light that can be detected. This strategy is highly sensitive and the expression of the reporter gene is stable. Another approach is based on the Cre-loxP system, which allows Cre-reporter cells that take up EVs released from cells that express Cre recombinase to be marked 118-122. EVs containing Cre mRNA can induce recombination in recipient cells carrying a fluorescent or enzymatic reporter gene. The main disadvantage of methods based on genetic modifications is that they are usually time consuming.

Only few studies testing the therapeutic efficacy of MSC-EVs have addressed the fate of MSC-EVs after their *in vivo* administration. In these reports mainly lipophilic dyes, with the affinity to cellular membranes such as PKH26, PKH67, CM-DiI, DiD or DiR have been used to label MSC-EVs (**Table 1**). The intravenously injected labeled MSC-EVs were detected in the injured organs already 1h after application, and remained in the injured tissue up to 7 days after administration 58,123-125. In the model of kidney inflammation in pigs, Eirin *et al*. found fluorescent signal of PKH26 labeled MSC-EVs even 4 weeks after administration 79. They demonstrated that only a fraction of fluorescent particles co-localized with CD9 exosomal marker, and none with CD63 marker, thus the authors interpreted this remaining fluorescent signal as MSC-EV fragments rather than intact vesicles. Only few reports used tissue/cell specific markers to more accurately define localization of the injected MSC-EVs. The MSC-EVs were found in tissue resident macrophages, but were also directly taken up by the cells from injured tissues 79,126,127. Lankford *et al*. were able to identify tissue resident macrophages targeted by MSC-EVs in a spine injury rat model, as anti- inflammatory M2 type 124. Interestingly, most of the studies in which the fate of MSC-EVs was followed found them localized preferentially to the site of injury, in contrast to the control animals where little signal of labeled MSC-EVs was detected in the tissue of interest 58,123,124,128,129. This specific MSC-EVs targeting was observed regardless of the type of the injury and organ studied. However, most of the studies also reported no injury related accumulation of MSC-EVs in organs such as liver, spleen and lungs, especially after IV or intraperitoneal (IP) injections 79,129,130. In other tissues the method of MSC-EVs administration did not significantly influence their destination. Importantly, in the two studies where they compared MSC-EVs efficacy after different ways of systemic delivery in liver injury mouse models, there was no significant difference in the beneficial effect of MSC-EVs on tissue recovery 128,130. This data is promising in the context of future clinical applications of MSC-EVs, as this would mean that the way of MSC-EVs delivery can be adjusted to lower the burden for the patient without affecting the efficacy and potency of the treatment 47,130. However, to be able to extrapolate this conclusion to conditions other than liver injury, similar research needs to be performed in animal models of different types of tissue injury. The targeting of MSC-EVs to specific organs seems to be dependent on the proteins present on their membranes. Bruno *et al*. demonstrated that treatment of MSC-EVs with trypsin abrogates their localization to the injured kidney and also to any other examined organs 58. In addition, the levels of trypsin-treated MSC-EVs remained constant in plasma, while the plasma levels of non-treated MSC-EVs markedly decreased in mice with acute kidney injury. The remaining question is whether this specific targeting of MSC-EVs to injured tissue is a unique feature of MSC-EVs alone, or are EVs derived from other cell types also capable of it. The evidence from *in vitro* studies in cells from neuronal system suggests that MSC-EVs are not an exception and that there exists a specificity in EVs targeting. Fitzner *et al*. showed that EVs derived from oligodendroglia are preferentially taken up by microglia and not by astrocytes, neurons or oligodendrocytes 131. However, to our knowledge no study addressed this matter *in vivo* in tissue repair. There are reports using EVs derived from fibroblasts to control for specificity of MSC-EVs effect in injured tissue 58,126,127,129,130,132-134. These studies show that fibroblasts do not have any beneficial effect on tissue regeneration, suggesting that indeed MSC-EVs are unique in the positive regulation of tissue repair. However, the distribution of fibroblast-derived EVs in different organs *in vivo* was not investigated in these reports.

As previously mentioned, the majority of studies have relied on lipophilic dyes to trace EV fate *in vivo*, however it is important to note their limitations, which may have an impact on the EV biology. Whilst these dyes can influence the normal performance of EVs, there is also a certain degree of aspecificity in the labeling as they can also associate with aggregates of lipoproteins or other lipid rich structures. In addition, their relatively short half-life is also restricting long term follow up. On the other hand, the dyes with longer half-life (PKH26) may remain in the *in vivo* system longer than the EVs themselves because they can be released from the EVs, which as a consequence will generate an aspecific signal. The latest advancements in the development of techniques that allow for the fate of EVs to be followed *in vivo,* such as those based on the use of Cre reporters or those using the new generation of dyes to label EVs, should help to elucidate the molecular mechanism by which MSC-EVs affect tissue repair 119,135. This may allow the design of MSC-EVs that can be more efficiently delivered to the tissue of interest.

The considerable effort has been already invested in engineering EVs to improve their targeting and enhance their use as drug delivery vehicles. The gene that encodes the targeting protein can be inserted into the donor cell that in turn secretes EVs containing the protein. In the report by Alvarez *et al.* the authors engineered dendritic cells to express Lamp2b, an EV membrane protein, fused to the neuron-specific RVG peptide and EVs produced by this cells carried this protein and had preferential binding to neurons. This way EVs after being loaded with siRNA of interest could effectively deliver it to the brain 136. Another study by Kooijman *et al.* used a method, in which the donor cells were designed to express a modified glycolipid that was fused with nanobodies to target specific cells. They were able to show that EVs carrying the anti-epidermal growth factor receptor (EGFR) nanobodies were specifically binding to EGFR-expressing tumor cells 137. These examples demonstrate that using similar strategies for MSC-EVs may significantly improve their therapeutics effects in tissue repair.

## Potency of different MSC-EV populations

The EVs secreted by different cell types are very heterogeneous. This is also the case for EVs released by MSCs 138,139. It would be very beneficial for a future clinical application of MSC-EVs if the subpopulation of MSC-EVs with the best therapeutic potential could be specifically identified. To our knowledge, only a handful of studies have attempted to address this through comparing the therapeutic effect of small EVs (pelleted at 100,000 g by ultracentrifugation), with larger EVs (pelleted at 18,000 g) 57,140,141. In the delayed-T hypersensitivity mouse model, small EVs were more efficient than larger EVs in reducing inflammation and they were also more potent in preventing mice from developing collagen-induced arthritis 57,140. Likewise, small EVs outperformed larger EVs in promoting kidney injury repair 142. In contrast, there was no difference in efficacy of small versus larger EVs in protecting mice from osteoarthritic damage in the collagenase-induced OA model. However, it should be noted that twice the number of larger EVs were used compared to smaller EVs, as measured by protein content 57. What accounts for the differences in the potency of different MSC-EV subsets still needs to be determined. One possibility is differences in membrane proteins decorating the distinct EV populations, which could translate to differential efficiency in targeting of the injured/diseased tissue. Another option could be the type of cargo carried by different EV subsets, which would make them immunomodulatory or have more regenerative potential. Indeed, Bruno *et al*. demonstrated that small EVs and larger EVs with different regenerative potency also have distinct molecular signatures regarding their miRNA, mRNA and protein content 141. The studies mentioned above illustrate distinct therapeutic potential of EV populations after relatively rough division to two subgroups. However, considering the span of sizes of vesicles falling into each category, these two groups do not represent fully homogeneous populations either 57,140,141. It is likely that there is also a difference in therapeutic efficacy of EVs subsets within each group.

## Impact of source and activation status of MSCs on therapeutic activity of MSC-EVs

An important question to address while considering the clinical application of MSC-EVs is the source of MSCs used for EV isolation. So far there has been a great diversity in the origin of MSCs used for isolation of EVs tested in pre-clinical animal models of different conditions. These included MSCs derived from umbilical cord, bone marrow, adipose tissue, synovium, Wharton jelly, menstrual blood, kidney, bowman's capsule and MSCs generated from embryonic and induced pluripotent stem cells (**Table 1**; 50,92,150-152,140,143-149). The majority of MSC-EVs isolated from MSC derived from all these different tissues had positive effects on tissue repair regardless of the type of injury. Only one study has reported no effects of EVs derived from MSCs generated from embryonic stem cells in chronic kidney disease model in rat 153. However, due to large differences in EV doses applied, isolation procedures or even the *in vivo* tissue injury models used, it is difficult to conclude from current studies whether a specific tissue source of MSCs is more favorable for the EV isolation with higher therapeutic potential. To our knowledge only two studies have compared the therapeutic efficacy of MSC-EVs derived from different tissue origin in the same *in vivo* experiment. Willis *et al*. have compared EVs isolated from bone marrow and Wharton jelly derived MSCs in a hyperoxia-induced Bronchopulmonary Dysplasia mouse model and reported equal efficacy of EVs isolated from both MSC types 134. In contrast, a study comparing EVs isolated from induced pluripotent stem cells-derived MSCs (iPSC-MSCs) and synovial membrane-derived MSCs demonstrated the superior therapeutic effect of EVs from iPSC-MSCs in OA mouse model 38.

Another relevant question is whether MSCs used for EV production need to be primed to increase therapeutic efficacy of generated vesicles. Only a few studies have compared the therapeutic potential of MSC-EVs isolated from MSC cultured under different conditions. Kilpinen *et al*. reported that umbilical cord derived MSCs (UC-MSCs) previously primed with interferon gamma (IFN-γ) possibly modified the intracellular biogenesis pathway of EVs and changed their cargo composition. As a consequence the therapeutic activity of these MSC-EVs was hampered in kidney injury 75. On the other hand, Ruppert *et al.* showed that treatment with EVs from bone marrow-derived MSCs (BM-MSCs) preconditioned with interferon gamma (IFN-γ) and tumor necrosis factor alpha (TNF-α), is at least as beneficial as using EVs from non-preconditioned BM-MSCs in rats with spinal cord injury 154. In specific aspects of the recovery from the injury, such as improvement of sensory function, EVs from cytokine-preconditioned MSCs had an even stronger beneficial effect than EVs from non-preconditioned cells. In a study by Cosenza *et al*. BM-MSCs were pretreated with TGF-β to uniquely study the chondroprotective function of MSC-EVs 57. The EVs from TGF-β -preconditioned MSCs protected mice from joint damage in a collagenase-induced OA model, but they were not compared with EVs from non-treated MSC, which makes it difficult to conclude whether MSCs preconditioning was indeed essential for the therapeutic effect. In a mouse model of cardiotoxin-induced muscle injury Lo Sicco *et al*. compared the anti-inflammatory properties of EVs isolated from adipose tissue derived MSCs (AD-MSCs) cultured under normoxic or hypoxic conditions 155. The authors found that EVs from hypoxic MSCs possess more effective anti- inflammatory properties than 'normoxic' EVs. In contrast, EVs from AD-MSCs starved for 12 hours under hypoxic conditions were less effective than EVs isolated from AD-MSCs cultured under normal conditions in improving survival and suppressing the inflammatory reaction in rats after induced sepsis syndrome 143. Collectively, preconditioning of MSCs to produce more therapeutically effective EVs may be relevant, but more research is necessary to define what type of MSCs pre-treatment is required and whether it should be tuned to the type of injury targeted by the EV-based therapy.

## Choice of pre-clinical model to test the therapeutic effect of MSC-EVs

The role of animal models in EV research in general has been recently covered by Reiner *et al*. in a review discussing development of best-practice models for the therapeutic use of EVs 156. Here, we highlight some aspects of pre-clinical application of MSC-EVs, which are especially relevant in tissue repair.

The choice of a good pre-clinical model to test the therapeutic efficacy of MSC- EVs in tissue regeneration is very important. Although completely mimicking the human condition is not realistic in any of the existing animal models, the pre-clinical testing should be performed in models, which most closely represent the human pathophysiology. It is also important to define in what stage of the disease the therapeutic effect is desired, and design the study accordingly. For example in conditions such as OA, the MSC-EVs could be applied at an earlier stage of the disease to prevent it from further development. On the other hand, it is also relevant to test whether MSC-EVs-based therapy is potent enough to attenuate fully developed OA. Thus, good timing of MSC-EV administration is crucial for testing their efficacy and potency. This might be of special importance in conditions where inflammation is a dominant component of the disease pathology such as autoimmune disorders, sepsis and GvHD. In many current studies testing the therapeutic potential of MSC-EVs, researchers have administered MSC-EVs before the induction of injury or before the full establishment of the disease (**Table 1**; 54,59,80,99,127,132,157-160). These studies report promising therapeutic effects of MSC-EVs, however translation of these findings to the clinic might require additional testing of MSC-EVs in more clinically relevant setup.

## Concluding remarks

The therapeutic potential of MSC-EVs is quite well documented in a variety of tissue injury models. Results from *in vivo* studies also indicate, that already after a single application, MSC-EVs are as efficient as their parental cells in promoting tissue regeneration. This is very promising for future clinical use of MSC-EVs and suggests that indeed MSC-EVs-based therapy may be a cheaper alternative to MSC-based treatments. However, the available data comparing the efficacy of MSC-EVs and their cellular counterparts need to be taken with a degree of caution considering limitations of the currently available EV quantification techniques. Similarly, more emphasis should be put on testing the optimal therapeutic doses of administered EVs. As highlighted above, current *in vivo* studies vary enormously in the amount of applied EVs. Also, there is still very limited evidence on the long-term effect of MSC-EVs. It is likely that for certain types of tissue injuries it will be unnecessary to administer MSC-EVs multiple times, whilst in other conditions multiple administrations may be required. This will increase the burden for patients and the cost of the therapy. Another important issue to address before the clinical introduction of MSC-EVs is their heterogeneity. Currently available data indicate that there are significant differences in the therapeutic activity of different MSC-EV entities. More research should be done in this direction to identify subpopulations of MSC-EVs with the highest therapeutic potential. Likewise, new strategies and more research (in the field of genomics, proteomic etc.) are needed to sufficiently classify and isolate EV sub-populations in a robust way with high accuracy and selectivity. Similarly, it will be essential to increase reproducibility of large scale preparation of EVs with high purity and defined therapeutic activity. This will also require the establishment of well-defined *in vitro* assays for quality control testing, which will in turn need to be tuned to the needs of the type of the condition treated with EVs. Current pre-clinical studies using labeled MSC-EVs report that the EVs target macrophages and the injured tissue of interest. This suggests that the beneficial effect of MSC-EVs in tissue repair is mediated not only by regulating the immune response around injured tissue, but also by direct interaction with the tissue. A more detailed investigation of this dual MSC-EVs activity can improve their targeting to the relevant tissues and increase their therapeutic efficacy. Importantly, acquiring more insights into the mechanism of action of MSC-EVs will help in defining their legal status and might improve their therapeutic activity, which is crucial for their future clinical application.

## Figures and Tables

**Table 1:** Summary of *in vivo* studies using MSC-derived EVs (2017-2019)

	Disease model	MSC origin	MSC source	EV isolation method	Controls used	Determination of EV size and concentration	Doses of EVs used	Injections	EV fate tracing	Disease status at time of first EV treatment	Effect	Ref
**Autoimmune disorders**	Sjögren's Syndrome (Mouse)	Human	iPSC and BM	UC 100k g	PBS iPSC-MSC BM-MSC	NTA	30ug (No cell equivalent given)	IV, 2x, 1 week interval	No	Early stage of disease	+	50
	Type 1 Diabetes (Mouse)	Human	BM	Chromatography on an anion exchange column	MSC Vehicle	Bradford, NTA and flow cytometry	15 x 10^9 or 30ug or 3ug (No cell equivalent given)	IV, 2x, 4 day interval	No	Preventive treatment	+	158
	Uveoretinitis (EAU) (Mouse)	Human	BM	Chromatography on an anion exchange column	PBS MSC	Bradford, NTA and flow cytometry	15 x 10^9 or 30 ug (No cell equivalent given)	IV, 1x	No	Preventive treatment	+	158
	Delayed-T hypersensitivity (DTH) (Mouse)	Mouse	BM	MP 18k g EVs 100k g	PBS MSC	Bradford, NTA and flow cytometry	250 ng EVs and MP (produced by 2.5x10^5 MSCs in 48 h)	IV, 1x	No	Preventive treatment	EVs and MP as efficient in preventing DTH	140
	Collagen Induced Arthritis (CIA) (Mouse)	Mouse	BM	UC MP 18k g EVs 100k g	PBS	Bradford, NTA and flow cytometry	3-fold or 2-fold less EVs or MPs, respectively, than the quantity produced by 1 x 10^6 MSCs in 48 h.	IV, 2x, 6 days interval	No	Preventive treatment	EVs more efficient than MP in preventing CIA	140
	Multiple sclerosos (MS) (Mouse)	Human	BM	UC MP 16,5k g EVs 120k g	PBS	Bradford, NTA and spectrophotometer	1.0 x 10^6 MSCs or 150 µg EVs	IV, 2x	DiR EVs in healthy and disease mice 3h and 24h after injection.	Established disease	+	161
**Bone disorders**	Osteogenesis imperfecta (Mouse)	Mouse	BM	UC 110k g	PBS *Ex vivo* controls: CM; EDCM; CM treated with ProtK	Bradford, NTA and flow cytometry	8.88 x10^8 particles/ mL (Equivalent of 1.09 x 10^6 MSC)	IV, 4x, 1 week interval	No	Established disease	+	147
**Brain disorder**	Traumatic brain injury (TBI) (Swine)	Human	BM	UC 110k g	Vehicle	qNano	10^13 particles/ 4ml bolus (No cell equivalent given)	IV, 5x, Different intervals between injections	No	Injection straight after injury (6h)	+	148
	Alzheimer's disease (Mouse)	Mouse	BM	PEG	PBS	Micro Bicinchoninic Acid assay	100ug (No cell equivalent given)	ICV, 7x, 2 day interval	No	Established disease	+	149
	Hypoxic ischemic (HI) induced neonatal brain insult (Rice- Vannucci model)	Human	BM	UC 100k g	PBS	NTA and flow cytometry	6 µL of EVs (1.25 x 10^9 particles/dose) (No cell equivalent given)	1x, Intranasal	No	Direct after injury	+	49
	Perinatal brain injury (Rat)	Human	WJ	UC 100k g	PBS	Electron microscopy, flow cytometry and Bicinchoninic acid protein assay	50 mg/kg (No cell equivalent given)	1x, Intranasal	PKH26 (*in vitro*)	Direct after injury	+	162
	Hypoxic ischemic encephalopathy (HIE) (fetal sheep)	Human	BM	PEG	0.9% sodium chloride	NTA and Tunable Resistive Pulse Sensing (TRPS)	Equivalent of 2x 10^7 MSC	2x at 1h and 4 days after injury through umbilical vessel catherer.	No	Early stages of injury	+	163
**OA and cartilage repair**	Collagenase induced OA (CIOA) (Mouse)	Human	iPSC and SV	Ultrafiltration	PBS	NTA, Tunable Resistive Pulse Sensing (TRPS) and TEM	8 µl EVs (1.0 x 10^10/ml) (No cell equivalent given)	IA injection on day 7,14, and 21.	No	Treatment 7,14 and 21 days after collagenase injection	+	38
	Surgery induced OA (Rat)	Human	SV	UC and sucrose/D_2_O cushion 100k g	PBS WT EVs Mir140overexpression EVs	DLS and TEM	100 μL; 1 x 10^11 EVs particles/mL (No cell equivalent given)	IA, 3x	DiO (*in vitro*)	Directly after injury	+	92
	Collagenase induced OA (CIOA) (Mouse)	Human	BM	UC 64k g and sucrose gradient UC at 100k g	PBS	NTA, TEM, flow cytometry, western blots	15 µl of 500 µg/ml (No cell equivalent given)	3x, No injection information	No	Treatment day 7 after collagenase injection	+	93
	Osteochondral defect (Rat)	Human	ESC	Ultrafiltration	PBS	Electrom microscopy and NanoOrange Protein Quantification	100ug (No cell equivalent given)	IA, 4x, 1 week interval	Alexa488 (*in vitro*)	Directly after injury	+	150
	Collagenase-induced OA (CIOA) (Mouse)	Mouse	BM	MP 20k g EVs 100k g	PBS MSC	Bradford, NTA and flow cytometry	500 ng MP; 250ng EVs (Equivalent of 48 h production by 2.5 × 10^5 BM-MSC)	IA, 1x	No	Treatment day 7 after collagenase injection	+	57
	Antigen- Induced Synovitis (Swine)	Porcine	BM	Ultrafiltration	PBS	Bradford, NTA and flow cytometry	500ug/injection (No cell equivalent given)	IA, 1x	No	Established disease	+	151
**GVHD**	Acute GVHD (Mouse)	Human	BM	Precipitation (Invitrogen kit)	PBS	Bradford and qNano	Approximately 1.6 × 10^7 particles containing 16 μg protein (EVs from 2 × 10^6 human BM-MSC/ kg body weight)	IV, 1x	No	Established disease	+	152
**Cardiac conditions**	Myocardial infarction (Mouse)	Mouse	BM	UC 100k g	PBS miRNA21 KO EVs	Bradford and flow cytometry	EVs (1 μl/g body weight; 0.5 μg/μl; 4.5×10^4 EVs per ug of protein (No cell equivalent given)	Injection into the pericardial sac, 1x	PKH26 (*in vitro*)	Preventive treatment	+	80
	I/R injury (Rat)	Rat	BM	Precipitation (Invitrogen kit)	Vehicle	BCA, TEM and qNano	5 ug (No cell equivalent given)	Injection directly into injury region, 1x	PKH 26 (*in vitro*)	Preventive treatment	+	159
**Kidney injury**	Glycerol induced AKI (Mouse)	Human	BM	UC EVs 100k g MVs 10k g	Vehicle	NTA, flow cytometry and TEM	16.5 x 10^7 particles per mouse (No cell equivalent given)	IV, 1x	No	3 days after injury induction	EVs but not MVs induce renal regeneration	141
	Kidney/cisplatin (Rat)	Human	CB	UC and sucrose/ D_2_O cushion 100k g	PBS EVs from fibroblasts	NTA and TEM	200 ug (No cell equivalent given)	Renal capsule injection, 1x	No	Preventive treatment	+	132
	High fat and high carbohydrate diet induced kidney inflammation (Swine)	Porcine	AD	UC 100k g	EVs from IL-10 KD cells	NTA and TEM	1 x 10^10 EVs (No cell equivalent given)	Intrarenal injection, 1x	PKH26 EVs in injured kidney at 2 days; also in liver, lung spleen. Little in hart and healthy kidney	Injection 6 weeks after induction of the injury	+	79
	Diabetic nephropathy (Mouse)	Human	BM	UC 100k G	EVs from fibroblasts	NTA	1 x 10^10 particles (No cell equivalent given)	IV, 5x, 1 week interval	No	Established disease	+	48
**Skeletal muscle injury**	Cardiotoxin-induced muscle injury (Mouse)	Human	AD	UC 100k g	PBS; EVs from MSC cultured under normoxia and hypoxia conditions	Bradford, flow cytometry and TEM	1ug (No cell equivalent given)	Injected into the right and left TA muscles, 2x, 4 day interval	PKH67 (*in vitro*)	Injection after injury	+	155
	Cardiotoxin-induced muscle injury (Mouse)	Human	AD	UC 200k g	PBS; whole secretome	TEM and NTA	100 µL EVs (Equivalent of 1x10^6 MSC)	1x IV injection through tail vein	PKH67 (*in vitro*)	Preventive treatment	+	164
**Sepsis**	Sepsis syndrome (Rat)	Rat	AD	?	EVs from apoptotic and healthy MSC	TEM and western blots	100ug (No cell equivalent given)	1x IV	No	Injection 3h after CLP	+	143
**Liver conditions**	Liver injury (Mouse)	Mouse	AD	ExoQuick	PBS	Bradford and TEM	40ug (No cell equivalent given)	Intrasplenic injection 16x, 3 days interval	cyc3-labelled pre-miRNA-transfected ADSC (*in vitro*)	Preventive treatment	EVs overexpresing miR-181-5p alleviate liver injury	99
	Cl4-induced liver injury (Mouse)	Human	CB	UC and sucrose/D_2_O cushion 100k g	PBS	NTA, flow cytometry and TEM	6 × 10^10 particles/kg, 1.2 × 10^11 particles/kg and 2.4 × 10^11 particles/kg (No cell equivalent given)	IV, 1x	CM-DiR EV in liver- (for a tumor model)	Injection after injury	+	144
	TAA-induced liver cirrhosis (Rat)	Human	ESC	UC 100k g	PBS MSC	DLS, flow cytometry, western blot and SEM	350ug; (No cell equivalent given)	Intrasplenicly	PKH-26 EVs in liver and in spleen	Established injury	+	165
	Induced lethal hepatic failure (D-galactosamine/ TNF-alpha) (Mouse)	Human and Mouse	BM	UC 100k g	PBS hMSC mMSC	NTA and TEM	2 x 10^8 - 2 x 10^10 particles per body (No cell equivalent given)	IP and IV	DiR EVs in healthy and injured liver 6 h after injection	Injection after injury	+	128
	Hepatic I/R injury (Mouse)	Mouse	BM	UC	PBS	NTA	2x10^10 particles (No cell equivalent given)	IP, IV, SC, orally (per os)	DiR EV distribution tested 6 h after injection	Preventive treatment	+	47
	CCl4-induced liver failure (Mouse)	Human	CB	UC and sucrose/D_2_O cushion 100k g	PBS EVs from fibroblasts	Bradford, NTA, flow cytometry and TEM	8, 16 or 32 mg/kg per body weight (No cell equivalent given)	IV and oral	CM-Dir EVs in injured and normal livers at 24 h post injection	24h after injury	+	130
	CCl4-induced liver failure (Mouse)	Human	CB	UC and sucrose/D_2_O cushion 100k g	PBS	NTA, flow cytometry and TEM	6x10^10, 1.2x10^11 or 2.4x10^11 particles/kg (No cell equivalent given)	IV or oral	CM-Dir	24h after injury	+	166
**Lung injury**	SwIV induced lung injury (Swine)	Swine	BM	UC (25k rpm)	DMEM	Micro-bicinchoninic acid protein assay, flow cytometry and TEM	80 μg/kg body weight. (EVs produced by 10 × 10^6 MSCs in 48 h)	Intratracheally, 1x	PKH26 (*In vitro*)	12 h after SwIV infection	+	167
	Acute liver injury (ALI) (Mouse)	Human	Placenta	UC 130k g	PBS, AIEgens (no EVs)	TEM, flow cytometry and NTA	100 µg EVs (No cell equivalent given)	Tail vein, 1x	AIEgens (DPA-SCP)	End-stage liver disease	+	168
	Lung ischemia-reperfusion injury (Mouse)	Human	CB	UC 100k g	MSC	Bradford, flow cytometry, NanoDrop UV spectrophotometer and NTA	Equivalent of 1x10^6 MSC	Intratracheally, 1x	No	Preventive treatment	+.	59
	Neonatal hyperoxic lung injury (Rat)	Human	CB	UC 100k g	PBS MSC EVs from fibroblasts	Bradford TEM and SEM	20ug (No cell equivalent given)	Intratracheally, 1x	PKH67; EVs 24h after injection In the lung and alveolar MQ	Established injury	+	126
	Hyperoxia-induced Bronchopulmonary Dysplasia (Mouse)	Human	WJ; BM	DC;TFF; OptiPrep™ cushion	EVs from WJ, BM MSC and fibroblasts	NTA and TEM	Equivalent of 0.5 x 10^6 MSC in 36 h	IV, 1x	DiL (*In vitro*)	Injection 4 days after start of hyperoxia conditions	+	134
**Spinal cord injury**	Spinal cord injury (Rat)	Rat	BM	UC 100k g	PBS PBS with DiR	Bradford, NTA and TEM	100 μg protein 2.5 ×10^9 EVs (No cell equivalent given)	Injection directly in spinal cord or IV	DiR EVs in spinal cord resident MQ2 3h and 24h after infusion; very rarely in intact spine	Established injury	Study only to show localization of the EVs	134
	Spinal Cord Contusion (Rat)	Human	BM	TFF system equipped with a Biomax 500 kDa (5 μm) Pellicon filter	PBS, EVs, EVs from TNF-alpha/INF-γ treated MSC	NTA and flow cytometry	1x1ml of 1×10^9 EV/ml (No cell equivalent given)	IV, 1x	No	3 hours after injury	+	154
	Spinal cord injury (Mouse)	Human	CB	UC 120k g	PBS	DLA, TEM and western blot	20ug and 200 ug (No cell equivalent given)	IV, 1x	No	30 min after injury	+	145
	Spinal cord injury (Rat)	Rat	BM	UC 110k g	PBS, EV-free CM	TEM and western blot	200 µL of EVs derived from 1 x 10^6 MSCs	Tail vein	PKH26	30 min after injury	+	169
**Tissue radiation**	Hematopoietic acute radiation syndrome (Mouse)	Human	BM	UC and sucrose/D_2_O cushion 100k g	PBS, DiD dye alone, fibroblast-derived EVs	NTA, TEM, flow cytometry, western blot	2x 10^8, 2x 10^9, 2x 10^10 one dose or 2x 10^9 three doses. (No cell equivalent given)	Tail vein	DiD	24 h after radiation	+	170

AD- adipose tissue; AKI- acute kidney injury; BM- bone marrow; CB- cord blood; CIA- collagen induced arthritis; CM- conditioned medium; DC- differential centrifugation; DLS- Dymanic light scattering; EDCM- EV depleted conditioned medium; ESC- embryonic stem cells; ICV- intracerebroventricular; iPSC- induced pluripotent stem cells; IP- intraperitoneal; IV- intravenous; MP- microparticles; NTA: Nanoparticle Tracking analysis; OA- osteoarthritis; SV- synovium; TBI- traumatic brain injury; TEM- Transmission Electron Microscopy; TFF- tangential flow filtration; UC- ultracentrifugation; WJ- Wharton jelly

**Table 2:** Strategies for EV labeling

Imaging technique	Labeling strategy	Labeling methods	Advantages	Disadvantages	Stability of the dye	Ref
Fluorescent (confocal microscopy)	Direct	DiR, DiD, PKH26, PKH67, (membrane bound)	Fast and simple and inexpensive protocol.	Nonspecific EV labeling because the dye releases from the EV. The half-life of the dye may be longer than the EV stability. High background to signal ratio. Dyes may affect the composition of EV membrane bilayer and EV functionality.	DiR: up to 4 weeks DiD: up to 24h PKH26: up to 84 days PKH67: up to 7 days: R18: up to 12 days	171, 172
		CFDA-SE Calcein AM (membrane permeable)	Can be used to discriminate between intact EVs and cell debris	Fluorescent dye can leak out of the cell/EV	CFDA-SE: robust stability, detectable up to 8 cell divisions Calcein AM: up to 36 hours	115-117
	Indirect	GFP, pH sensitive GFP, RFP, dTomato (Fusions with Palm, CD63 etc.)	Cell type specific	Requires genetic modification and is time intensive.	Expression is stable	173-175
		Cre-recombinase based system	Accurate analysis of the physiological EV uptake.	Time consuming and requires genetic modification. Not quantitative.	Stable reporter gene.	118-122, 176
Bioluminescence (light microscopy with CCD camera)	Indirect	Gluc, GlucB, Rluc	Highly sensitive	Requires genetic modification and is time intensive. The substrates (e.g. coelenterazine) can be toxic.	Stable reporter.	177-179
Nuclear imaging (SPECT, PET)	Direct	111 In-oxine, 99mTc- HMPAO, 99mTc-tricarbonyl, 125I-biotin derivatives	Stable and highly quantitative. High tissue penetration depth. Used in the clinic	Requires knowledge with radioactivity-based detection. EVs are lost during labeling.	HMPAO: half-life 37 min 111 In-oxine: half-life 67 hours. 99mTc-tricarbonyl: half-life 6 hours. 125I-biotin derivatives: half-life 2.7 min.	177,180-182
Magnetic resonance	Direct	USPIO	Labeling does not affect the size and biodistribution of EVs.	The sensitivity of USPIOs is low therefore large amounts of EVs is needed.	Half-life: 24 h.	183

## References

[1] Le Blanc K, Mougiakakos D (2012). Multipotent mesenchymal stromal cells and the innate immune system. Nat Rev Immunol.

[2] Dominici M, Le Blanc K, Mueller I (2006). Minimal criteria for defining multipotent mesenchymal stromal cells. The International Society for Cellular Therapy position statement. Cytotherapy.

[3] Noiseux N, Gnecchi M, Lopez-Ilasaca M (2006). Mesenchymal stem cells overexpressing Akt dramatically repair infarcted myocardium and improve cardiac function despite infrequent cellular fusion or differentiation. Mol Ther.

[4] Iso Y, Spees JL, Serrano C (2007). Multipotent human stromal cells improve cardiac function after myocardial infarction in mice without long-term engraftment. Biochem Biophys Res Commun.

[5] Swart JF, de Roock S, Hofhuis FM (2014). Mesenchymal stem cell therapy in proteoglycan induced arthritis. Ann Rheum Dis.

[6] de Windt TS, Vonk LA, Slaper-Cortenbach ICM (2017). Allogeneic Mesenchymal Stem Cells Stimulate Cartilage Regeneration and Are Safe for Single-Stage Cartilage Repair in Humans upon Mixture with Recycled Autologous Chondrons. Stem Cells.

[7] Lee RH, Pulin AA, Seo MJ (2009). Intravenous hMSCs Improve Myocardial Infarction in Mice because Cells Embolized in Lung Are Activated to Secrete the Anti-inflammatory Protein TSG-6. Cell Stem Cell.

[8] Toma C, Wagner WR, Bowry S, Schwartz A, Villanueva F (2009). Fate of culture-expanded mesenchymal stem cells in the microvasculature: in vivo observations of cell kinetics. Circ Res.

[9] Bruno S, Grange C, Collino F (2012). Microvesicles derived from mesenchymal stem cells enhance survival in a lethal model of acute kidney injury. PLoS One.

[10] Lai RC, Arslan F, Lee MM (2010). Addendum to Exosome secreted by MSC reduces myocardial ischemia/reperfusion injury. Stem Cell Research.

[11] Zhang B, Yin Y, Lai RC, Tan SS, Choo ABH, Lim SK (2013). Mesenchymal Stem Cells Secrete Immunologically Active Exosomes. Stem Cells Dev.

[12] Zhang B, Wang M, Gong A (2014). HucMSC-exosome mediated -Wnt4 signaling is required for cutaneous wound healing. Stem Cells.

[13] Zaborowski MP, Balaj L, Breakefield XO, Lai CP (2015). Extracellular Vesicles: Composition, Biological Relevance, and Methods of Study. Bioscience.

[14] van Niel G, D'Angelo G, Raposo G (2018). Shedding light on the cell biology of extracellular vesicles. Nat Rev Mol Cell Biol.

[15] Raposo G, Stoorvogel W (2013). Extracellular vesicles: Exosomes, microvesicles, and friends. J Cell Biol.

[16] Van Niel G, D'Angelo G, Raposo G (2018). Shedding light on the cell biology of extracellular vesicles. Nature Reviews Molecular Cell Biology.

[17] Théry C, Witwer KW, Aikawa E (2018). Minimal information for studies of extracellular vesicles 2018 (MISEV2018): a position statement of the International Society for Extracellular Vesicles and update of the MISEV2014 guidelines. J Extracell Vesicles.

[18] Witwer KW, Van Balkom BWM, Bruno S (2019). Defining mesenchymal stromal cell (MSC)-derived small extracellular vesicles for therapeutic applications. J Extracell Vesicles.

[19] Lötvall J, Hill AF, Hochberg F (2014). Minimal experimental requirements for definition of extracellular vesicles and their functions: A position statement from the International Society for Extracellular Vesicles. J Extracell Vesicles.

[20] Valadi H, Ekström K, Bossios A, Sjöstrand M, Lee JJ, Lötvall JO (2007). Exosome-mediated transfer of mRNAs and microRNAs is a novel mechanism of genetic exchange between cells. Nat Cell Biol.

[21] Williams C, Royo F, Aizpurua-Olaizola O (2018). Glycosylation of extracellular vesicles: current knowledge, tools and clinical perspectives. J Extracell Vesicles.

[22] Yang X-X, Sun C, Wang L, Guo X-L (2019). New insight into isolation, identification techniques and medical applications of exosomes. J Control Release.

[23] Rupert DLM, Claudio V, Lässer C, Bally M (2017). Methods for the physical characterization and quantification of extracellular vesicles in biological samples. Biochimica et Biophysica Acta - General Subjects.

[24] Hartjes TA, Mytnyk S, Jenster GW, van Steijn V, van Royen ME (2019). Extracellular vesicle quantification and characterization: Common methods and emerging approaches. Bioengineering.

[25] Ramirez MI, Amorim MG, Gadelha C (2018). Technical challenges of working with extracellular vesicles. Nanoscale.

[26] Wang W, Luo J, Wang S (2018). Recent Progress in Isolation and Detection of Extracellular Vesicles for Cancer Diagnostics. Advanced Healthcare Materials.

[27] Boriachek K, Islam MN, Möller A (2018). Biological Functions and Current Advances in Isolation and Detection Strategies for Exosome Nanovesicles. Small.

[28] Gudbergsson JM, Johnsen KB, Skov MN, Duroux M (2016). Systematic review of factors influencing extracellular vesicle yield from cell cultures. Cytotechnology.

[29] K.W (2013). W, E.I. B, L.T. B, et al. Standardization of sample collection, isolation and analysis methods in extracellular vesicle research. J Extracell Vesicles.

[30] Théry C, Amigorena S, Raposo G, Clayton A (2006). Isolation and Characterization of Exosomes from Cell Culture Supernatants and Biological Fluids. Curr Protoc Cell Biol.

[31] Poliakov A, Spilman M, Dokland T, Amling CL, Mobley JA (2009). Structural heterogeneity and protein composition of exosome-like vesicles (prostasomes) in human semen. Prostate.

[32] Momen-Heravi F (2017). Isolation of Extracellular Vesicles by Ultracentrifugation. Methods Mol Biol.

[33] Grubisic Z, Rempp P, Benoit H (2011). A Universal Calibration for Gel Permeation Chromatography. Rubber Chem Technol.

[34] Mol EA, Goumans MJ, Doevendans PA, Sluijter JPG, Vader P (2017). Higher functionality of extracellular vesicles isolated using size-exclusion chromatography compared to ultracentrifugation. Nanomedicine Nanotechnology, Biol Med.

[35] Monguió-Tortajada M, Roura S, Gálvez-Montón C (2017). Nanosized UCMSC-derived extracellular vesicles but not conditioned medium exclusively inhibit the inflammatory response of stimulated T cells: Implications for nanomedicine. Theranostics.

[36] Witwer KW, Buzás EI, Bemis LT (2013). Standardization of sample collection, isolation and analysis methods in extracellular vesicle research. J Extracell Vesicles.

[37] Xue C, Shen Y, Li X (2018). Exosomes Derived from Hypoxia-Treated Human Adipose Mesenchymal Stem Cells Enhance Angiogenesis Through the PKA Signaling Pathway. Stem Cells Dev.

[38] Zhu Y, Wang Y, Zhao B (2017). Comparison of exosomes secreted by induced pluripotent stem cell-derived mesenchymal stem cells and synovial membrane-derived mesenchymal stem cells for the treatment of osteoarthritis. Stem Cell Res Ther.

[39] Bari E, Perteghella S, Catenacci L (2019). Freeze-dried and GMP-compliant pharmaceuticals containing exosomes for acellular mesenchymal stromal cell immunomodulant therapy. Nanomedicine.

[40] Klymiuk MC, Balz N, Elashry MI, Heimann M, Wenisch S, Arnhold S (2019). Exosomes isolation and identification from equine mesenchymal stem cells 06 Biological Sciences 0601 Biochemistry and Cell Biology. BMC Vet Res.

[41] Heinemann ML, Ilmer M, Silva LP (2014). Benchtop isolation and characterization of functional exosomes by sequential filtration. J Chromatogr A.

[42] Nordin JZ, Lee Y, Vader P (2015). Ultrafiltration with size-exclusion liquid chromatography for high yield isolation of extracellular vesicles preserving intact biophysical and functional properties. Nanomedicine.

[43] Weng Y, Sui Z, Shan Y (2016). Effective isolation of exosomes with polyethylene glycol from cell culture supernatant for in-depth proteome profiling. Analyst.

[44] McBride JD, Rodriguez-Menocal L, Guzman W, Candanedo A, Garcia-Contreras M, Badiavas E V (2017). Bone Marrow Mesenchymal Stem Cell-Derived CD63 ^+^ Exosomes Transport Wnt3a Exteriorly and Enhance Dermal Fibroblast Proliferation, Migration, and Angiogenesis In Vitro. Stem Cells Dev.

[45] Lane RE, Korbie D, Trau M, Hill MM (2017). Purification Protocols for Extracellular Vesicles. Methods Mol Biol.

[46] Dragovic RA, Gardiner C, Brooks AS (2011). Sizing and phenotyping of cellular vesicles using Nanoparticle Tracking Analysis. Nanomedicine Nanotechnology, Biol Med.

[47] Haga H, Yan IK, Borrelli DA (2017). Extracellular vesicles from bone marrow-derived mesenchymal stem cells protect against murine hepatic ischemia/reperfusion injury. Liver Transplant.

[48] Grange C, Tritta S, Tapparo M (2019). Stem cell-derived extracellular vesicles inhibit and revert fibrosis progression in a mouse model of diabetic nephropathy. Sci Rep.

[49] Sisa C Kholia S Naylor J Mesenchymal stromal cell derived extracellular vesicles reduce hypoxia-ischaemia induced perinatal injury Front Physiol 2019 190282. doi: 10.3389/fphys.2019.00282 10.3389/fphys.2019.00282 PMC6433879 30941062

[50] Hai B, Shigemoto-Kuroda T, Zhao Q, Lee RH, Liu F (2018). Inhibitory Effects of iPSC-MSCs and Their Extracellular Vesicles on the Onset of Sialadenitis in a Mouse Model of Sjögren's Syndrome. Stem Cells Int.

[51] Sitar S, Kejžar A, Pahovnik D (2015). Size Characterization and Quantification of Exosomes by Asymmetrical-Flow Field-Flow Fractionation. Anal Chem.

[52] Vonk LA, van Dooremalen SFJ, Liv N (2018). Mesenchymal Stromal/stem Cell-derived Extracellular Vesicles Promote Human Cartilage Regeneration *In Vitro*. Theranostics.

[53] Price B (1999). John J. Bozzola and Lonnie D. Electron Microscopy, Second Edition, Russell. Sudbury, MA: Jones and Bartlett Publishers.

[54] Lai RC, Arslan F, Lee MM (2010). Exosome secreted by MSC reduces myocardial ischemia/reperfusion injury. Stem Cell Res.

[55] Tatischeff I, Larquet E, Falcón-Pérez JM, Turpin PY, Kruglik SG (2012). Fast characterisation of cell-derived extracellular vesicles by nanoparticles tracking analysis, cryo-electron microscopy, and Raman tweezers microspectroscopy. J Extracell Vesicles.

[56] Kim D, Nishida H, An SY, Shetty AK, Bartosh TJ, Prockop DJ (2016). Chromatographically isolated CD63+CD81+ extracellular vesicles from mesenchymal stromal cells rescue cognitive impairments after TBI. Proc Natl Acad Sci U S A.

[57] Cosenza S, Ruiz M, Toupet K, Jorgensen C, Noël D (2017). Mesenchymal stem cells derived exosomes and microparticles protect cartilage and bone from degradation in osteoarthritis. Sci Rep.

[58] Bruno S, Grange C, Deregibus MC (2009). Mesenchymal Stem Cell-Derived Microvesicles Protect Against Acute Tubular Injury. J Am Soc Nephrol.

[59] Stone ML, Zhao Y, Robert Smith J (2017). Mesenchymal stromal cell-derived extracellular vesicles attenuate lung ischemia-reperfusion injury and enhance reconditioning of donor lungs after circulatory death. Respir Res [Internet].

[60] Sze SK, de Kleijn DP V, Lai RC (2007). Elucidating the Secretion Proteome of Human Embryonic Stem Cell-derived Mesenchymal Stem Cells. Mol Cell Proteomics.

[61] Qazi TH, Mooney DJ, Duda GN, Geissler S (2017). Biomaterials that promote cell-cell interactions enhance the paracrine function of MSCs. Biomaterials.

[62] Su N, Gao PL, Wang K, Wang JY, Zhong Y, Luo Y (2017). Fibrous scaffolds potentiate the paracrine function of mesenchymal stem cells: A new dimension in cell-material interaction. Biomaterials.

[63] Phan J, Kumar P, Hao D, Gao K, Farmer D, Wang A (2018). Engineering mesenchymal stem cells to improve their exosome efficacy and yield for cell-free therapy. J Extracell Vesicles.

[64] Fang S, Xu C, Zhang Y (2016). Umbilical Cord-Derived Mesenchymal Stem Cell-Derived Exosomal MicroRNAs Suppress Myofibroblast Differentiation by Inhibiting the Transforming Growth Factor-β/SMAD2 Pathway During Wound Healing. Stem Cells Transl Med.

[65] Baglio SR, Rooijers K, Koppers-Lalic D (2015). Human bone marrow- and adipose-mesenchymal stem cells secrete exosomes enriched in distinctive miRNA and tRNA species. Stem Cell Res Ther.

[66] Eirin A, Riester SM, Zhu XY (2014). MicroRNA and mRNA cargo of extracellular vesicles from porcine adipose tissue-derived mesenchymal stem cells. Gene.

[67] Kim HS, Choi DY, Yun SJ (2012). Proteomic analysis of microvesicles derived from human mesenchymal stem cells. J Proteome Res.

[68] Angulski ABB, Capriglione LG, Batista M (2017). The Protein Content of Extracellular Vesicles Derived from Expanded Human Umbilical Cord Blood-Derived CD133+ and Human Bone Marrow-Derived Mesenchymal Stem Cells Partially Explains Why both Sources are Advantageous for Regenerative Medicine. Stem Cell Rev Reports.

[69] Anderson JD, Johansson HJ, Graham CS (2016). Comprehensive proteomic analysis of mesenchymal stem cell exosomes reveals modulation of angiogenesis via nuclear factor-kappaB signaling. Stem Cells.

[70] La Greca A, Solari C, Furmento V (2018). Extracellular vesicles from pluripotent stem cell-derived mesenchymal stem cells acquire a stromal modulatory proteomic pattern during differentiation. Exp Mol Med.

[71] Lai RC, Tan SS, Teh BJ (2012). Proteolytic Potential of the MSC Exosome Proteome: Implications for an Exosome-Mediated Delivery of Therapeutic Proteasome. Int J Proteomics.

[72] Eirin A, Zhu XY, Puranik AS Comparative proteomic analysis of extracellular vesicles isolated from porcine adipose tissue-derived mesenchymal stem/stromal cells. Sci Rep. 2016.

[73] van Balkom BWM, Gremmels H, Giebel B, Lim SK (2019). Proteomic Signature of Mesenchymal Stromal Cell-Derived Small Extracellular Vesicles. Proteomics.

[74] Salomon C, Ryan J, Sobrevia L (2013). Exosomal signaling during hypoxia mediates microvascular endothelial cell migration and vasculogenesis. PLoS One.

[75] Kilpinen L, Impola U, Sankkila L (2013). Extracellular membrane vesicles from umbilical cord blood-derived MSC protect against ischemic acute kidney injury, a feature that is lost after inflammatory conditioning. J Extracell vesicles.

[76] Zhu LP, Tian T, Wang JY (2018). Hypoxia-elicited mesenchymal stem cell-derived exosomes facilitates cardiac repair through miR-125b-mediated prevention of cell death in myocardial infarction. Theranostics.

[77] Zhang B, Shen L, Shi H (2016). Exosomes from Human Umbilical Cord Mesenchymal Stem Cells: Identification, Purification, and Biological Characteristics. Stem Cells Int.

[78] Harrell CR, Fellabaum C, Jovicic N, Djonov V, Arsenijevic N, Volarevic V (2019). Molecular Mechanisms Responsible for Therapeutic Potential of Mesenchymal Stem Cell-Derived Secretome. Cells.

[79] Eirin A, Zhu X-Y, Puranik AS (2017). Mesenchymal stem cell-derived extracellular vesicles attenuate kidney inflammation. Kidney Int.

[80] Luther KM, Haar L, McGuinness M (2018). Exosomal miR-21a-5p mediates cardioprotection by mesenchymal stem cells. J Mol Cell Cardiol.

[81] Hu S, Park J, Liu A (2018). Mesenchymal Stem Cell Microvesicles Restore Protein Permeability Across Primary Cultures of Injured Human Lung Microvascular Endothelial Cells. Stem Cells Transl Med.

[82] Zhao X, Wu X, Qian M, Song Y, Wu D, Zhang W (2018). Knockdown of TGF-β1 expression in human umbilical cord mesenchymal stem cells reverts their exosome-mediated EMT promoting effect on lung cancer cells. Cancer Lett.

[83] Wang H, Zheng R, Chen Q, Shao J, Yu J, Hu S (2017). Mesenchymal stem cells microvesicles stabilize endothelial barrier function partly mediated by hepatocyte growth factor (HGF). Stem Cell Res Ther.

[84] Liu Y, Zou R, Wang Z, Wen C, Zhang F, Lin F (2018). Exosomal KLF3-AS1 from hMSCs promoted cartilage repair and chondrocyte proliferation in osteoarthritis. Biochem J.

[85] Ma J, Zhao Y, Sun L (2017). Exosomes Derived from Akt -Modified Human Umbilical Cord Mesenchymal Stem Cells Improve Cardiac Regeneration and Promote Angiogenesis via Activating Platelet-Derived Growth Factor D. Stem Cells Transl Med.

[86] Zhang Y, Hao Z, Wang P (2019). Exosomes from human umbilical cord mesenchymal stem cells enhance fracture healing through HIF-1α-mediated promotion of angiogenesis in a rat model of stabilized fracture. Cell Prolif.

[87] Kang K, Ma R, Cai W (2015). Exosomes Secreted from CXCR4 Overexpressing Mesenchymal Stem Cells Promote Cardioprotection via Akt Signaling Pathway following Myocardial Infarction. Stem Cells Int.

[88] Ji W, Jiang W, Li M, Li J, Li Z (2019). miR-21 deficiency contributes to the impaired protective effects of obese rat mesenchymal stem cell-derived exosomes against spinal cord injury. Biochimie.

[89] Yu B, Kim HW, Gong M (2015). Exosomes secreted from GATA-4 overexpressing mesenchymal stem cells serve as a reservoir of anti-apoptotic microRNAs for cardioprotection. Int J Cardiol.

[90] Hnatiuk AP, Ong SG, Olea FD (2016). Allogeneic mesenchymal stromal cells overexpressing mutant human Hypoxia-inducible factor 1-α (HIF1-α) in an ovine model of acute myocardial infarction. J Am Heart Assoc.

[91] O'Brien KP, Khan S, Gilligan KE (2018). Employing mesenchymal stem cells to support tumor-targeted delivery of extracellular vesicle (EV)-encapsulated microRNA-379. Oncogene.

[92] Tao S-C, Yuan T, Zhang Y-L, Yin W-J, Guo S-C, Zhang C-Q (2017). Exosomes derived from miR-140-5p-overexpressing human synovial mesenchymal stem cells enhance cartilage tissue regeneration and prevent osteoarthritis of the knee in a rat model. Theranostics.

[93] Mao G, Zhang Z, Hu S (2018). Exosomes derived from miR-92a-3poverexpressing human mesenchymal stem cells enhance chondrogenesis and suppress cartilage degradation via targeting WNT5A. Stem Cell Res Ther.

[94] Tao S-C, Guo S-C, Li M, Ke Q-F, Guo Y-P, Zhang C-Q (2017). Chitosan Wound Dressings Incorporating Exosomes Derived from MicroRNA-126-Overexpressing Synovium Mesenchymal Stem Cells Provide Sustained Release of Exosomes and Heal Full-Thickness Skin Defects in a Diabetic Rat Model. Stem Cells Transl Med.

[95] Sharif S, Ghahremani MH, Soleimani M (2018). Delivery of Exogenous miR-124 to Glioblastoma Multiform Cells by Wharton's Jelly Mesenchymal Stem Cells Decreases Cell Proliferation and Migration, and Confers Chemosensitivity. Stem Cell Rev Reports.

[96] Li X, Liu L, Yang J (2016). Exosome Derived From Human Umbilical Cord Mesenchymal Stem Cell Mediates MiR-181c Attenuating Burn-induced Excessive Inflammation. EBioMedicine.

[97] Lou G, Yang Y, Liu F (2017). MiR-122 modification enhances the therapeutic efficacy of adipose tissue-derived mesenchymal stem cells against liver fibrosis. J Cell Mol Med.

[98] Katakowski M, Buller B, Zheng X (2013). Exosomes from marrow stromal cells expressing miR-146b inhibit glioma growth. Cancer Lett.

[99] Qu Y, Zhang Q, Cai X (2017). Exosomes derived from miR-181-5p-modified adipose-derived mesenchymal stem cells prevent liver fibrosis *via* autophagy activation. J Cell Mol Med.

[100] Chen L, Lu F bin, Chen D zhi (2018). BMSCs-derived miR-223-containing exosomes contribute to liver protection in experimental autoimmune hepatitis. Mol Immunol.

[101] Zhang Y, Chopp M, Liu XS (2017). Exosomes Derived from Mesenchymal Stromal Cells Promote Axonal Growth of Cortical Neurons. Mol Neurobiol.

[102] Chen Y, Zhao Y, Chen W (2017). MicroRNA-133 overexpression promotes the therapeutic efficacy of mesenchymal stem cells on acute myocardial infarction. Stem Cell Res Ther.

[103] Yu B, Gong M, Wang Y (2013). Cardiomyocyte Protection by GATA-4 Gene Engineered Mesenchymal Stem Cells Is Partially Mediated by Translocation of miR-221 in Microvesicles. PLoS One.

[104] Shen H, Yao X, Li H (2018). Role of Exosomes Derived from miR-133b Modified MSCs in an Experimental Rat Model of Intracerebral Hemorrhage. J Mol Neurosci.

[105] Lang FM, Hossain A, Gumin J (2018). Mesenchymal stem cells as natural biofactories for exosomes carrying miR-124a in the treatment of gliomas. Neuro Oncol.

[106] Wang B, Yao K, Huuskes BM (2016). Mesenchymal stem cells deliver exogenous MicroRNA-let7c via exosomes to attenuate renal fibrosis. Mol Ther.

[107] Chaubey S, Thueson S, Ponnalagu D (2018). Early gestational mesenchymal stem cell secretome attenuates experimental bronchopulmonary dysplasia in part via exosome-associated factor TSG-6. Stem Cell Res Ther.

[108] Shen B, Liu J, Zhang F (2016). CCR2 Positive Exosome Released by Mesenchymal Stem Cells Suppresses Macrophage Functions and Alleviates Ischemia/Reperfusion-Induced Renal Injury. Stem Cells Int.

[109] Reis M, Mavin E, Nicholson L, Green K, Dickinson AM, Wang XN (2018). Mesenchymal stromal cell-derived extracellular vesicles attenuate dendritic cell maturation and function. Front Immunol.

[110] Wang X, Gu H, Qin D (2015). Exosomal MIR-223 Contributes to Mesenchymal Stem Cell-Elicited Cardioprotection in Polymicrobial Sepsis. Sci Rep.

[111] Martin-Rufino JD, Espinosa-Lara N, Osugui L, Sanchez-Guijo F (2019). Targeting the Immune System With Mesenchymal Stromal Cell-Derived Extracellular Vesicles: What Is the Cargo's Mechanism of Action?. Frontiers in Bioengineering and Biotechnology.

[112] Hu GW, Li Q, Niu X (2015). Exosomes secreted by human-induced pluripotent stem cell-derived mesenchymal stem cells attenuate limb ischemia by promoting angiogenesis in mice. Stem Cell Res Ther.

[113] Zhu YG, Feng XM, Abbott J (2014). Human mesenchymal stem cell microvesicles for treatment of Escherichia coli endotoxin-induced acute lung injury in mice. Stem Cells.

[114] Jiang M, Wang H, Jin M (2018). Exosomes from MiR-30d-5p-ADSCs Reverse Acute Ischemic Stroke-Induced, Autophagy-Mediated Brain Injury by Promoting M2 Microglial/Macrophage Polarization. Cell Physiol Biochem.

[115] Hao S, Bai O, Li F, Yuan J, Laferte S, Xiang J (2006). Mature dendritic cells pulsed with exosomes stimulate efficient cytotoxic T-lymphocyte responses and antitumour immunity. Immunology.

[116] Keller S, König A-K, Marmé F (2009). Systemic presence and tumor-growth promoting effect of ovarian carcinoma released exosomes. Cancer Lett.

[117] Pospichalova V, Svoboda J, Dave Z (2015). Simplified protocol for flow cytometry analysis of fluorescently labeled exosomes and microvesicles using dedicated flow cytometer. J Extracell Vesicles.

[118] Ridder K, Sevko A, Heide J (2015). Extracellular vesicle-mediated transfer of functional RNA in the tumor microenvironment. Oncoimmunology.

[119] Ridder K, Keller S, Dams M (2014). Extracellular vesicle-mediated transfer of genetic information between the hematopoietic system and the brain in response to inflammation. PLoS Biol.

[120] Frühbeis C, Fröhlich D, Kuo WP (2013). Neurotransmitter-Triggered Transfer of Exosomes Mediates Oligodendrocyte-Neuron Communication. PLoS Biol.

[121] Zomer A, Steenbeek SC, Maynard C, Van Rheenen J (2016). Studying extracellular vesicle transfer by a Cre-loxP method. Nat Protoc.

[122] Sterzenbach U, Putz U, Low LH, Silke J, Tan SS, Howitt J (2017). Engineered Exosomes as Vehicles for Biologically Active Proteins. Mol Ther.

[123] Choi HY, Lee HG, Kim BS (2015). Mesenchymal stem cell-derived microparticles ameliorate peritubular capillary rarefaction via inhibition of endothelial-mesenchymal transition and decrease tubulointerstitial fibrosis in unilateral ureteral obstruction. Stem Cell Res Ther.

[124] Lankford KL, Arroyo EJ, Nazimek K, Bryniarski K, Askenase PW, Kocsis JD (2018). Intravenously delivered mesenchymal stem cell-derived exosomes target M2-type macrophages in the injured spinal cord. Sabaawy HE, Ed. PLoS One.

[125] Yang J, Liu X-X, Fan H (2015). Extracellular Vesicles Derived from Bone Marrow Mesenchymal Stem Cells Protect against Experimental Colitis via Attenuating Colon Inflammation, Oxidative Stress and Apoptosis. Camussi G, Ed. PLoS One.

[126] Ahn SY, Park WS, Kim YE (2018). Vascular endothelial growth factor mediates the therapeutic efficacy of mesenchymal stem cell-derived extracellular vesicles against neonatal hyperoxic lung injury. Exp Mol Med.

[127] Tamura R, Uemoto S, Tabata Y (2016). Immunosuppressive effect of mesenchymal stem cell-derived exosomes on a concanavalin A-induced liver injury model. Inflamm Regen.

[128] Haga H, Yan IK, Takahashi K, Matsuda A, Patel T (2017). Extracellular Vesicles from Bone Marrow-Derived Mesenchymal Stem Cells Improve Survival from Lethal Hepatic Failure in Mice. Stem Cells Transl Med.

[129] Gatti S, Bruno S, Deregibus MC (2011). Microvesicles derived from human adult mesenchymal stem cells protect against ischaemia-reperfusion-induced acute and chronic kidney injury. Nephrol Dial Transplant.

[130] Yan Y, Jiang W, Tan Y (2017). hucMSC Exosome-Derived GPX1 Is Required for the Recovery of Hepatic Oxidant Injury. Mol Ther.

[131] Fitzner D, Schnaars M, Van Rossum D (2011). Selective transfer of exosomes from oligodendrocytes to microglia by macropinocytosis. J Cell Sci.

[132] Wang B, Jia H, Zhang B (2017). Pre-incubation with hucMSC-exosomes prevents cisplatin-induced nephrotoxicity by activating autophagy. Stem Cell Res Ther.

[133] Zhou Y, Xu H, Xu W (2013). Exosomes released by human umbilical cord mesenchymal stem cells protect against cisplatin-induced renal oxidative stress and apoptosis in vivo and in vitro. Stem Cell Res Ther.

[134] Willis GR, Fernandez-Gonzalez A, Anastas J (2018). Mesenchymal Stromal Cell Exosomes Ameliorate Experimental Bronchopulmonary Dysplasia and Restore Lung Function through Macrophage Immunomodulation. Am J Respir Crit Care Med.

[135] Zomer A, Maynard C, Verweij FJ (2015). In vivo imaging reveals extracellular vesicle-mediated phenocopying of metastatic behavior. Cell.

[136] Alvarez-Erviti L, Seow Y, Yin H, Betts C, Lakhal S, Wood MJ (2011). Delivery of siRNA to the mouse brain by systemic injection of targeted exosomes. Nat Biotechnol.

[137] Kooijmans SAA, Aleza CG, Roffler SR, van Solinge WW, Vader P, Schiffelers RM (2016). Display of GPI-anchored anti-EGFR nanobodies on extracellular vesicles promotes tumour cell targeting. J Extracell Vesicles.

[138] Lázaro-Ibáñez E, Lässer C, Shelke GV (2019). DNA analysis of low- and high-density fractions defines heterogeneous subpopulations of small extracellular vesicles based on their DNA cargo and topology. J Extracell Vesicles.

[139] Lai RC, Tan SS, Yeo RWY (2016). MSC secretes at least 3 EV types each with a unique permutation of membrane lipid, protein and RNA. J Extracell Vesicles.

[140] Cosenza S, Toupet K, Maumus M (2018). Mesenchymal stem cells-derived exosomes are more immunosuppressive than microparticles in inflammatory arthritis. Theranostics.

[141] Bruno S, Tapparo M, Collino F (2017). Renal Regenerative Potential of Different Extracellular Vesicle Populations Derived from Bone Marrow Mesenchymal Stromal Cells. Tissue Eng Part A.

[142] Bruno S, Grange C, Collino F (2012). Microvesicles Derived from Mesenchymal Stem Cells Enhance Survival in a Lethal Model of Acute Kidney Injury. Câmara NOS, Ed. PLoS One.

[143] Chang C-L, Sung P-H, Chen K-H (2018). Adipose-derived mesenchymal stem cell-derived exosomes alleviate overwhelming systemic inflammatory reaction and organ damage and improve outcome in rat sepsis syndrome. Am J Transl Res.

[144] Jiang W, Tan Y, Cai M (2018). Human Umbilical Cord MSC-Derived Exosomes Suppress the Development of CCl _4_ -Induced Liver Injury through Antioxidant Effect. Stem Cells Int.

[145] Sun G, Li G, Li D (2018). hucMSC derived exosomes promote functional recovery in spinal cord injury mice via attenuating inflammation. Mater Sci Eng C.

[146] Börger V, Bremer M, Ferrer-Tur R (2017). Mesenchymal Stem/Stromal Cell-Derived Extracellular Vesicles and Their Potential as Novel Immunomodulatory Therapeutic Agents. Int J Mol Sci.

[147] Otsuru S, Desbourdes L, Guess AJ (2018). Extracellular vesicles released from mesenchymal stromal cells stimulate bone growth in osteogenesis imperfecta. Cytotherapy.

[148] Williams AM, Dennahy IS, Bhatti UF (2018). Mesenchymal Stem Cell-Derived Exosomes Provide Neuroprotection and Improve Long-Term Neurologic Outcomes in a Swine Model of Traumatic Brain Injury and Hemorrhagic Shock. J Neurotrauma. 2018; neu.

[149] Wang S-S, Jia J, Wang Z (2018). Mesenchymal Stem Cell-Derived Extracellular Vesicles Suppresses iNOS Expression and Ameliorates Neural Impairment in Alzheimer's Disease Mice. J Alzheimer's Dis.

[150] Zhang S, Chuah SJ, Lai RC, Hui JHP, Lim SK, Toh WS (2018). MSC exosomes mediate cartilage repair by enhancing proliferation, attenuating apoptosis and modulating immune reactivity. Biomaterials.

[151] Casado JG, Blázquez R, Vela FJ, Álvarez V, Tarazona R, Sánchez-Margallo FM (2017). Mesenchymal Stem Cell-Derived Exosomes: Immunomodulatory Evaluation in an Antigen-Induced Synovitis Porcine Model. Front Vet Sci.

[152] Fujii S, Miura Y, Fujishiro A (2018). Graft-Versus-Host Disease Amelioration by Human Bone Marrow Mesenchymal Stromal/Stem Cell-Derived Extracellular Vesicles Is Associated with Peripheral Preservation of Naive T Cell Populations. Stem Cells.

[153] van Koppen A, Joles JA, van Balkom BWM (2012). Human embryonic mesenchymal stem cell-derived conditioned medium rescues kidney function in rats with established chronic kidney disease. Dussaule J-C, Ed. PLoS One.

[154] Ruppert KA, Nguyen TT, Prabhakara KS (2018). Human Mesenchymal Stromal Cell-Derived Extracellular Vesicles Modify Microglial Response and Improve Clinical Outcomes in Experimental Spinal Cord Injury. Sci Rep.

[155] Lo Sicco C, Reverberi D, Balbi C (2017). Mesenchymal Stem Cell-Derived Extracellular Vesicles as Mediators of Anti-Inflammatory Effects: Endorsement of Macrophage Polarization. Stem Cells Transl Med.

[156] Reiner AT, Witwer KW, van Balkom BWM (2017). Concise Review: Developing Best-Practice Models for the Therapeutic Use of Extracellular Vesicles. Stem Cells Transl Med.

[157] Arslan F, Lai RC, Smeets MB (2013). Mesenchymal stem cell-derived exosomes increase ATP levels, decrease oxidative stress and activate PI3K/Akt pathway to enhance myocardial viability and prevent adverse remodeling after myocardial ischemia/reperfusion injury. Stem Cell Res.

[158] Shigemoto-Kuroda T, Oh JY, Kim D (2017). MSC-derived Extracellular Vesicles Attenuate Immune Responses in Two Autoimmune Murine Models: Type 1 Diabetes and Uveoretinitis. Stem Cell Reports.

[159] Liu L, Jin X, Hu C-F, Li R, Zhou Z, Shen C-X (2017). Exosomes Derived from Mesenchymal Stem Cells Rescue Myocardial Ischaemia/Reperfusion Injury by Inducing Cardiomyocyte Autophagy Via AMPK and Akt Pathways. Cell Physiol Biochem.

[160] Wang L, Gu Z, Zhao X (2016). Extracellular Vesicles Released from Human Umbilical Cord-Derived Mesenchymal Stromal Cells Prevent Life-Threatening Acute Graft-Versus-Host Disease in a Mouse Model of Allogeneic Hematopoietic Stem Cell Transplantation. Stem Cells Dev.

[161] Riazifar M, Mohammadi MR, Pone EJ (2019). Stem Cell-Derived Exosomes as Nanotherapeutics for Autoimmune and Neurodegenerative Disorders. ACS Nano.

[162] Thomi G, Surbek D, Haesler V, Joerger-Messerli M, Schoeberlein A (2019). Exosomes derived from umbilical cord mesenchymal stem cells reduce microglia-mediated neuroinflammation in perinatal brain injury. Stem Cell Res Ther.

[163] Gussenhoven R, Klein L, Ophelders D (2019). Annexin A1 as Neuroprotective Determinant for Blood-Brain Barrier Integrity in Neonatal Hypoxic-Ischemic Encephalopathy. J Clin Med.

[164] Mitchell R, Mellows B, Sheard J (2019). Secretome of adipose-derived mesenchymal stem cells promotes skeletal muscle regeneration through synergistic action of extracellular vesicle cargo and soluble proteins. Stem Cell Res Ther.

[165] Mardpour S, Hassani S-N, Mardpour S (2018). Extracellular vesicles derived from human embryonic stem cell-MSCs ameliorate cirrhosis in thioacetamide-induced chronic liver injury. J Cell Physiol.

[166] Jiang W, Cai M, Zhao T (2018). Human umbilical cord MSC-derived exosomes suppress the development of CCl4-induced liver injury through antioxidant effect. Stem Cells Int.

[167] Khatri M, Richardson LA, Meulia T (2018). Mesenchymal stem cell-derived extracellular vesicles attenuate influenza virus-induced acute lung injury in a pig model. Stem Cell Res Ther.

[168] Cao H, Yue Z, Gao H (2019). In Vivo Real-Time Imaging of Extracellular Vesicles in Liver Regeneration via Aggregation-Induced Emission Luminogens. ACS Nano.

[169] Lu Y, Zhou Y, Zhang R (2019). Bone Mesenchymal Stem Cell-Derived Extracellular Vesicles Promote Recovery Following Spinal Cord Injury via Improvement of the Integrity of the Blood-Spinal Cord Barrier. Front Neurosci.

[170] Wen S, Dooner M, Papa E (2019). Biodistribution of mesenchymal stem cell-derived extracellular vesicles in a radiation injury bone marrow murine model. Int J Mol Sci.

[171] GRANGE C, TAPPARO M, BRUNO S (2014). Biodistribution of mesenchymal stem cell-derived extracellular vesicles in a model of acute kidney injury monitored by optical imaging. Int J Mol Med.

[172] Yoshida K, Tsuda M, Matsumoto R (2019). Exosomes containing ErbB2/CRK induce vascular growth in premetastatic niches and promote metastasis of bladder cancer. Cancer Sci.

[173] Lai CP, Kim EY, Badr CE (2015). Visualization and tracking of tumour extracellular vesicle delivery and RNA translation using multiplexed reporters. Nat Commun.

[174] Verweij FJ, Revenu C, Arras G (2019). Live Tracking of Inter-organ Communication by Endogenous Exosomes In Vivo. Dev Cell.

[175] Verweij FJ, Bebelman MP, Jimenez CR (2018). Quantifying exosome secretion from single cells reveals a modulatory role for GPCR signaling. J Cell Biol.

[176] Tkach M, Théry C (2016). Communication by Extracellular Vesicles: Where We Are and Where We Need to Go. Cell.

[177] Takahashi Y, Nishikawa M, Shinotsuka H (2013). Visualization and in vivo tracking of the exosomes of murine melanoma B16-BL6 cells in mice after intravenous injection. J Biotechnol.

[178] Lai CP, Mardini O, Ericsson M (2014). Dynamic biodistribution of extracellular vesicles in vivo using a multimodal imaging reporter. ACS Nano.

[179] Gangadaran P, Li XJ, Lee HW (2017). A new bioluminescent reporter system to study the biodistribution of systematically injected tumor-derived bioluminescent extracellular vesicles in mice. Oncotarget.

[180] Audenaert K, Jansen HML, Otte A (2003). Imaging of mild traumatic brain injury using 57Co and 99mTc HMPAO SPECT as compared to other diagnostic procedures. Med Sci Monit.

[181] Morishita M, Takahashi Y, Nishikawa M (2015). Quantitative analysis of tissue distribution of the B16BL6-derived exosomes using a streptavidin-lactadherin fusion protein and Iodine-125-Labeled biotin derivative after intravenous injection in mice. J Pharm Sci.

[182] Imai T, Takahashi Y, Nishikawa M (2015). Macrophage-dependent clearance of systemically administered B16BL6-derived exosomes from the blood circulation in mice. J Extracell Vesicles.

[183] Ahn BC Requisites for successful theranostics with radionuclide-based reporter gene imaging. Journal of Drug Targeting. 204; 22: 295-303.

